# RUNX1-BMP2 promotes vasculogenic mimicry in laryngeal squamous cell carcinoma via activation of the PI3K-AKT signaling pathway

**DOI:** 10.1186/s12964-024-01605-x

**Published:** 2024-04-12

**Authors:** Qingwen Zhu, Xinyu Zhang, Fei Lu, Siyu Miao, Chunyang Zhang, Zhenzhen Liu, Zejun Gao, Meihao Qi, Xiaogang An, Panling Geng, Sufang Wang, Hongbo Ren, Fugen Han, Ruyue Zhang, DingJun Zha

**Affiliations:** 1 Department of Otorhinolaryngology Head and Neck Surgery, Xijing Hospital, The Air Force Military Medical University, Xi’an, China; 2 Department of Otorhinolaryngology Head and Neck Surgery, Children’s Hospital Affiliated to Zhengzhou University, Zhengzhou, China; 3 Department of Oncology, The First Affiliated Hospital of Zhengzhou University, Zhengzhou, China

**Keywords:** Laryngeal squamous cell carcinoma, Vasculogenic mimicry, Bone morphogenetic protein 2, Metastasis, Runt-related transcription factor 1

## Abstract

**Background:**

Laryngeal squamous cell carcinoma (LSCC) is one of the most common malignant tumors of the head and neck. Vasculogenic mimicry (VM) is crucial for tumor growth and metastasis and refers to the formation of fluid channels by invasive tumor cells rather than endothelial cells. However, the regulatory mechanisms underlying VM during the malignant progression of LSCC remain largely unknown.

**Methods:**

Gene expression and clinical data for LSCC were obtained from the TCGA and Gene GEO (GSE27020) databases. A risk prediction model associated with VM was established using LASSO and Cox regression analyses. Based on their risk scores, patients with LSCC were categorized into high- and low-risk groups. The disparities in immune infiltration, tumor mutational burden (TMB), and functional enrichment between these two groups were examined. The core genes in LSCC were identified using the machine learning (SVM-RFE) and WGCNA algorithms. Subsequently, the involvement of bone morphogenetic protein 2 (BMP2) in VM and metastasis was investigated both in vitro and in vivo. To elucidate the downstream signaling pathways regulated by BMP2, western blotting was performed. Additionally, ChIP experiments were employed to identify the key transcription factors responsible for modulating the expression of BMP2.

**Results:**

We established a new precise prognostic model for LSCC related to VM based on three genes: *BMP2*, *EPO*, and *AGPS*. The ROC curves from both TCGA and GSE27020 validation cohorts demonstrated precision survival prediction capabilities, with the nomogram showing some net clinical benefit. Multiple algorithm analyses indicated *BMP2* as a potential core gene. Further experiments suggested that BMP2 promotes VM and metastasis in LSCC. The malignant progression of LSCC is promoted by BMP2 via the activation of the PI3K-AKT signaling pathway, with the high expression of BMP2 in LSCC resulting from its transcriptional activation by runt-related transcription factor 1 (RUNX1).

**Conclusion:**

BMP2 predicts poor prognosis in LSCC, promotes LSCC VM and metastasis through the PI3K-AKT signaling pathway, and is transcriptionally regulated by RUNX1. BMP2 may be a novel, precise, diagnostic, and therapeutic biomarker of LSCC.

**Supplementary Information:**

The online version contains supplementary material available at 10.1186/s12964-024-01605-x.

## Introduction

Laryngeal squamous cell carcinoma (LSCC) is a type of head and neck malignant tumor that originates in the laryngeal mucosal epithelium. Its incidence and mortality rates are increasing annually [1, 2]. LSCC is susceptible to tumor invasion, metastasis, and chemotherapy resistance, which are the major factors associated with poor prognosis in patients [3]. Vasculogenic mimicry (VM) is an emerging concept in cancer biology [4, 5]. Unlike traditional angiogenesis, in which tumors stimulate the growth of blood vessels in tumor tissues, VM represents a distinct mechanism by which aggressive cancer cells form directly into vessel-like structures, allowing them to independently access essential nutrients and oxygen [6, 7]. Several molecular pathways and factors are involved in VM formation, including epithelial-mesenchymal transition (EMT), hypoxia, and cancer stem cells (CSCs) [8]. Clinically, VM is usually assessed in patient samples by identifying vessels that are positive for periodic acid-Schiff (PAS) staining but negative for CD31 or CD34, as indicated by immunohistochemistry (IHC) analysis. PAS stains the basement membrane, whereas CD31 and CD34 are endothelial cell markers [9]. In addition, as VE-cadherin plays a pivotal role in VM formation, it thus serves as a biomarker of VM. Of note, VM is associated with poor prognosis, increased metastasis, and decreased patient survival rates in various cancers [10–13]. Recent studies have shown that VM density is higher in patients with LSCC with local lymph node metastases and those with a higher histopathological grade [14]. This was consistent with the findings of Lin et al., which indicated that VM was positively associated with higher pTNM stages and lymph node metastases [15]. Moreover, VM appears to drive distant metastasis in breast cancer while facilitating the entry of red blood cells and nutrients into the tumor tissue [16]. With the growing interest in VM as a new target for tumor therapy, clarifying its impact on prognosis will enhance its potential as a biomarker. However, the role of VM-associated genes in LSCC prognosis remains unclear. Furthermore, exploring new biological markers and molecular mechanisms affecting VM and metastasis in LSCC is of the utmost importance.

Bone morphogenetic proteins (BMPs) are members of the transforming growth factor-β (TGF-β) superfamily [17]. They have similar sequences and share the same BMP receptors, operating through a common downstream signaling pathway known as the BMP signaling pathway [18]. They participate in the regulation of various biological processes, such as proliferation, apoptosis, embryonic development, tumor formation, EMT, and metastasis [19, 20]. BMP2 is a vital component of the BMP family and is involved in the complex signaling networks of various regulatory proteins. It not only plays a crucial role in bones but is also involved in various important biological processes in cancer, such as the regulation of EMT and cancer stem cells (CSCs) [21]. Accordingly, BMP2 antagonists were reported to block the angiogenic effects of BMP2 in breast cancer and melanoma cells [22, 23]. Recent studies have reported the inverse correlation of BMP2 with the tumor suppressor gene PTEN, as BMP2 downregulates the expression of PTEN [24]. BMP2 is highly overexpressed in nonsmall cell lung cancer (NSCLC) and associated with tumor staging and metastasis [25]. These findings suggested that BMP2 is a viable therapeutic target for cancer and a novel biomarker for assessing treatment efficacy. However, the role and detailed molecular mechanisms of action of BMP2 in most cancers, including LSCC, remain unclear.

This study aimed to identify reliable biomarkers associated with LSCC prognosis. We investigated the role of BMP2 in VM and LSCC metastasis and further elucidated the upstream and downstream regulatory mechanisms of BMP2. Our study offers novel insights into BMP2 as a new prognostic biomarker and potential therapeutic target for the future clinical treatment of LSCC.

## Methods

### Collecting and preprocessing data

RNA sequencing data of 111 LSCC samples and 12 normal laryngeal tissues were obtained from the TCGA (The Cancer Genome Atlas) database (https://portal.gdc.cancer.gov). Clinical information and RNA sequencing data of LSCC samples were obtained from the GSE27020 and GSE51985 Gene Expression Datasets (https://www.ncbi.nlm.nih.gov/geo/). Gene expression data were normalized using the "SangerBox" tool (http://sangerbox.com/) before further analysis.

### Identification of Differentially Expressed Genes (DEGs)

In total, 334 VM-related genes were obtained from the GeneCards Database (https://www.genecards.org). Using the "DESeq2" package, differentially expressed genes (DEGs) related to apoptosis were identified in the 111 LSCC and 12 normal samples. The threshold was set at *P* < 0.05, and log2 fold change (FC) > 0.585. Using the R "survival" and "survminer" packages, univariate analysis was conducted on DEGs related to overall survival (OS). The correlation of prognostic genes was further analyzed using the R packages "igraph," "psych," "reshape2," and "RColorBrewer."

### Construction and validation of a VM-related genes(VMRGs) prognostic model

Based on 334 VMRGs, we screened 141 VM-related differential genes (VMDEGs) from normal and LSCC samples and used these 141 differential genes to construct a prognostic model. LASSO Cox regression analysis and least absolute shrinkage were applied to 141 VMDEGs to construct a prognostic model. Cross-validation (CV) algorithm was used for model selection criteria and validation. A minimum mean cross-validated error rule and a 10-fold cross-validation approach were used to select the penalization parameter λ in LASSO. We calculated the risk score using the following formula:

$$\mathrm{Risk}\;\mathrm{score}=\sum_{K-1}^ncoef\left({mRNA}^K\right)\;\times\;\exp\;r\left({mRNA}^K\right)$$

where n is the number of risk genes, coef is the regression coefficient in the multivariate Cox regression analysis (VMRGs), and expr is the expression of the risk-associated genes (BMP2, EPO, and AGPS). The median risk score was used to divide patients with LSCC into high- and low-risk groups, followed by OS assessment. The R package "timeROC" was used to plot time-dependent receiver operating characteristic (ROC) curves and areas under the curves (AUCs). We validated the model using an LSCC cohort from the GEO database to enhance its credibility. We then determined whether the risk score contributed to overall survival (OS) and progression-free survival (PFS) in the cohort using multivariate and univariate Cox regression analyses. Covariates included age, sex, and TNM stage.

### Functional enrichment and Tumor Mutational Burden (TMB) analyses

Functional enrichment analyses (GO, KEGG, and GSEA) were performed using the "clusterProfiler" package as previously reported [26]. In addition, Gene Set Variation Analysis (GSVA) was conducted using the "GSVA" R package to further identify differentially enriched KEGG pathways. Using the R package "MAfTools," we calculated the TMB of each tumor based on the number of mutations per million bases calculated from the somatic mutation data.

### Cell culture

HaCaT, TU212, and TU686 cells were purchased from iCell Biotechnology Co., LTD. (Shanghai, China). All three cells had STR testing certificates (Fig. S1). According to Mycoplasma Detection Kit (#G238; abm, Canada), all cell lines were negative for mycoplasma (Fig. S2). TU212 is a laryngeal squamous cell carcinoma cell line and TU686 is a hypopharyngeal carcinoma cell line. TU212 and TU686 cells were cultured in DMEM (Gibco, Carlsbad, CA, USA) supplemented with 10% fetal bovine serum (Gibco) and 1% penicillin/streptomycin (Sigma-Aldrich) at 37 °C in a humidified atmosphere containing 5% CO_2_. Prior to subsequent experiments, cells were treated with MK2206 (5um), a highly selective Akt inhibitor, for 12 h.

### Western Blotting (WB) and immunohistochemistry (IHC)

Total protein was extracted using a protein extraction buffer (#GK10023; GLPBIO, Montclair, CA, USA) containing protease (#GK10014; GLPBIO) and phosphatase (#GK10013; GLPBIO) inhibitors. Protein samples were then separated by sodium dodecyl sulfate–polyacrylamide gel electrophoresis and transferred to PVDF membranes (Millipore, Billerica, MA, USA), which were then blocked by incubation with 5% BSA for 1 h. Subsequently, membranes were incubated with a blocking buffer containing the primary antibody overnight at 4 °C, washed in TBST, and then incubated in TBST containing the HRP-conjugated secondary antibodies (M21008, Abmart, Shanghai, China). A Bio-Rad ECL Western Blotting Detection System (Bio-Rad, Hercules, CA, USA) was used for visualization. For measuring molecular weight, PierceTM unstained protein MW Marker (Thermo Fisher, 26610; UElandy, P6110, Suzhou, China) was used. The antibodies used were as follows: BMP2 (dilution 1:2000, #66383–1-Ig; ProteinTech, Wuhan, China), ACTB (dilution 1:3000, #81115–1-RR; ProteinTech), RUNX1 (dilution 1:1000, #25315–1-AP; ProteinTech), VE-cadherin (dilution 1:500, #A22659; Abclonal, Wuhan, China), N-cadherin (dilution 1:500, #A19083; Abclonal), E-cadherin (dilution 1:500, #A3044; Abclonal), AKT (dilution 1:1000, #4691S; CST, Danvers, MA, USA), p-AKT (dilution 1:1000, #4060S; CST), p-PTEN (dilution 1:1000, #9551S; CST), and p-PDK1(dilution 1:1000, #3438S; CST).

An LSCC tissue microarray (HN049La01) was purchased from Zhongke Guanghua Intelligent Biotechnology Co., Ltd. (Xian, China). IHC was performed as previously described [27], using an immunohistochemistry kit (Zhongshan Goldenbridge Biotechnology, Beijing, China). Tumor tissue sections were incubated with the primary antibody overnight at 4 °C. Next, sections were rinsed using phosphate-buffered saline (PBS) and then incubated with horseradish peroxidase (HRP)-labeled IgG for 60 min, followed by the addition of DAB (Zhongshan Goldenbridge). Finally, sections were counterstained with hematoxylin and eosin (H&E). The staining intensities of sections were independently assessed by two pathologists. The following antibodies were used: BMP2 (dilution 1:50), RUNX1 (dilution 1:150), VE-ca (dilution 1:150), N-ca (dilution 1:150), and E-ca (dilution 1:150).

### Plasmids and shRNA constructs

The BMP2-overexpressing plasmid (pCDNA3.1-BMP2), shRNAs targeting BMP2 or RUNX1, and their respective negative control RNAs were purchased from Tsingke Biotech Co., Ltd. (Beijing, China). Transfection was performed using Lipofectamine 2000 (Invitrogen, Carisbad, CA) with a plasmid-to-transfection reagent ratio of 1:2. Using the pLKO.1-CMV-LUC-PURO vector, Lv-shBMP2 and Lv-NC cells were constructed. Lentiviral transfection was performed according to the manufacturer’s instructions. Fresh complete medium was added to cultures 2 d after infection. Cells were selected using puromycin (3 μg/mL, Invitrogen) for 7 d to obtain stably transfected cells.

The shRNA sequences used were as follows: shBMP2-1: 5′-CCGGCCGGAGATTCTTCTTTAATTTCTCGAGAAATTAAAGAAGAATCTCCGGTTTTTT-3′; shBMP2-2: 5′-CCGGGATCATCTGAACTCCACTAATCTCGAGATTAGTGGAGTTCAGATGATCTTTTTT-3′; and shRUNX1: 5′-CCGGCCTCGAAGACATCGGCAGAAACTCGAGTTTCTGCCGATGTCTTCGAGGTTTTTT-3′.

### Quantitative real-time PCR (qRT-PCR)

TRIzol reagent (Invitrogen) was used to extract total RNA, which was then reverse transcribed into cDNA using a reverse transcription system (Takara, Tokyo, Japan). qRT-PCR was performed using the SYBR Green Mastermix (Takara) with the CFX96 Touch qRT-PCR System (Bio-Rad). For relative quantification, the mRNA levels of target genes were normalized to those of β-actin. The primer sequences used were as follows: β-actin: forward sequence, 5′-CTCCATCCTGGCCTCGCTGT-3′; reverse sequence, 3′-GCTGTCACCTTCACCGTTCC-5′. BMP2: forward sequence, 5′-CGCTGCCCAGAGGACTTC-3′; reverse sequence, 3′-GACTTTAGCGGTCTCGGAGC-5′. RUNX1: forward sequence, 5′-CTGCCCATCGCTTTCAAGGT-3′; reverse sequence, 3′-GCCGAGTAGTTTTCATCATTGCC-5′.

### Wound healing and Transwell assays

The migratory ability of cells was evaluated using a wound healing assay. A 6-well plate was seeded with shBMP2-transfected TU686 and TU212 cells. After reaching 100% confluence, the cell monolayer was wounded with a 10 μL pipette tip and then cultured in a basic medium. At specified times, the wound area was quantified using the ImageJ software(Invitrogen (Carisbad, CA). Each experiment was repeated thrice. Two types of Transwell assays were used: migration and invasion. Using Transwell inserts (#3422; Corning, NY, USA) with an 8-µm-pore size, we added 5 × 10^4^ cells resuspended in basic medium to the upper chamber, whereas 10% fetal bovine serum was added to the lower chamber. Transwell invasion assays involved the use of 60 µL Matrigel (#356234; Corning) precoated onto the upper membrane. Following an 18 h migration or 36 h invasion assay, cells were fixed with 4% paraformaldehyde(P8430, Solarbio, Beijing, China) and stained with 0.1% crystal violet (G1061, Solarbio). Digital images of membranes were obtained by capturing three random fields in each chamber.

### Spheroid invasion assay

For the spheroid invasion assay, spheroids were formed using a previously described method [26]. After 3d, they were embedded in a gel prepared using a 1:1 mixture of Matrigel and DMEM/F-12 medium. After 2 d, the total invasion area was divided by the central spheroid area, and measurements were taken using ImageJ.

### Matrigel tube formation and plug assays

The Matrigel tube formation assay was conducted to evaluate the ability of TU212 and TU686 cells to form vessel-like structures, which is a key event in VM. After dissolving it at 4 °C, 60 μL Matrigel was added to each well in a 96-well plate and allowed to polymerize at 37 °C for 1 h. Subsequently, 4 × 10^4^ LSCC cells were resuspended in 100 μL DMEM/F12 medium and incubated at 37 °C in 5% CO_2_. The tube formation ability of cells was observed every 3 h. The Cytation5 Cell Imaging Multimode Reader (BioTek, Winooski, VT, USA) was used for image acquisition.

Nude mice (4-week-old) were used for in vivo Matrigel plug assays. Nude mice were subcutaneously injected in the axilla of the forelimb with TU212 cells (5 × 10^6^) mixed with Matrigel (0.2 mL). On the seventh day, mice were euthanized, and the Matrigel plugs were excised. The density of newly formed VM was measured by H&E staining of histological sections.

### BALB/c nude mice and zebrafish tumor models

TU212 cells were transfected with Lv-luc-shBMP2 or empty vector, and stable transfectants were selected. Next, 2 × 10^6^ luciferase-labeled TU212 cells in 100 µL DMEM medium were injected into the tail veins of male nude mice aged 5 weeks (n = 4 per group). Over a 5-week course, D-luciferin (150 mg/kg, #GC43496; GLPBIO, Montclair, CA, USA) was intraperitoneally injected into mice, and whole-body photon flux was measured weekly using a Xenogen IVIS imaging system (Caliper Life Sciences, Mountain View, CA, USA) to monitor lung metastasis. To verify the inhibitory effect of the *BMP2* knockdown on LSCC growth in vivo, TU212 cells (5 × 10^7^) were resuspended in 200 μL DMEM medium and injected subcutaneously into the right axilla of nude mice. Tumor volume was measured every 3 d. Tumor volume was calculated using the following equation: tumor volume (mm^3^) = (tumor width)^2^ × tumor length / 2. At 21 d after tumor implantation, mice were euthanized, and xenografts were removed, fixed, weighed, photographed, and preserved.

The zebrafish tumor model was developed as previously described [22]. Animals were housed at the Zebrafish Centre of the First Affiliated Laboratory of the Air Force Medical University. Using a microinjection system, TU212 cells (200 cells/5 nL) were injected into the yolk sac cavity of zebrafish embryos after being labelled with 2 g/mL DiI (V228885, ThermoFisher, Waltham, MA, USA).

### Immunofluorescent (IF) staining

Cells cultured in 10-mm glass bottom dishes were fixed with 4% paraformaldehyde and blocked with 5% BSA at 25 °C for 30 min. Fixed cells were incubated overnight at 4 °C with primary antibodies against E-cadherin (1:200); N-cadherin (1:200); VE-cadherin (1:200, #ab313632; Abcam); BMP2 (1:50); and RUNX1 (1:100). After washing, cells were incubated with fluorescence-labeled secondary antibodies (1:200; Invitrogen) at 37 °C for 30 min. Nuclei were counterstained with Hoechst solution (1:1000, #33258; Beyotime). Images were captured using a FV3000 confocal fluorescence microscope (Olympus, Center Valley, PA, USA).

### Chromatin immunoprecipitation (ChIP)

A ChIP assay kit (17–295; Merck, Darmstadt, Germany) was used according to the manufacturer’s instructions. Briefly, cells were crosslinked with 1% formaldehyde and sonicated using the Covaris M220 system (Covaris, Woburn, USA) to generate DNA fragments of 100–500 bp in length. After preclearing, the supernatant was incubated with antibodies against RUNX1 (10 g rabbit IgG, ab272456; Abcam) or isotype control antibody (2 g rabbit IgG, ab172730; Abcam). PCR was conducted using primers targeting the *BMP2* promoter binding sites (RUNX1 set1, forward sequence: 5′-ATTTCCAGCCTGCTGTTTTCTT-3′; reverse sequence: 3′-CCACTCCCTGCTCTCAAAGGA-5′ and RUNX1 set2, forward sequence: 5′-ACATATTAACCGAAATGTGGCCC-3′; reverse sequence: 3′-GGAAAATTAAAAGAAAACAGCAGGC-5′). DNA isolated from the total nuclear extract was used as a control. PCR products were run on 2% agarose gel.

### Dual-luciferase reporter assay.

The dual-luciferase reporter system (Dual-Luciferase Reporter Assay; Promega, Madison, WI, USA) was used. The sequence of the *BMP2* promoter region (from the transcription start site: -2000 bp to + 100 bp) was obtained from the UCSC database (Table S1). Transcription factors associated with *BMP2* were predicted using the AnimalTFDB (AnimalTFDB4 (hust.edu.cn)), GTRD (http://gtrd20-06.biouml.org/), and JASPAR (https://jaspar.genereg.net/) databases. Potential binding sites for RUNX1 within the *BMP2* promoter region were predicted using JASPAR (https://jaspar.genereg.net/). The pcDNA3.1-RUNX1 and pcDNA3.1-NC vectors were constructed, and the wild type (WT) and mutant (MT) sequences (Table S2) of the promoter region were cloned into the pGL3 vector (TsingKe Biotech Co., Ltd.). The promoter-specific and pcDNA3.1 constructs were cotransfected into TU212 cells with the control Renilla luciferase construct PRL-TK, using the Hieff TransTM reagent (YEASEN, Shanghai, China). Luciferase and Renilla luciferase assays were performed for normalization.

### Statistical analysis

All data are expressed as the mean ± standard deviation. Student’s *t*-test was conducted using the GraphPad Prism (GraphPad Prism v9.0; GraphPad Software, USA) and R software(R version 4.1.1, R Core Team Vienna, Austria). One-way analysis of variance was used to compare data among multiple groups. A *P* < 0.05 was considered statistically significant. Statistical significance was defined as follows: *P < 0.05, **P < 0.01, and ***P < 0.001.

## Results

### Construction of the VM-related gene prognostic model

**Fig. 1:**
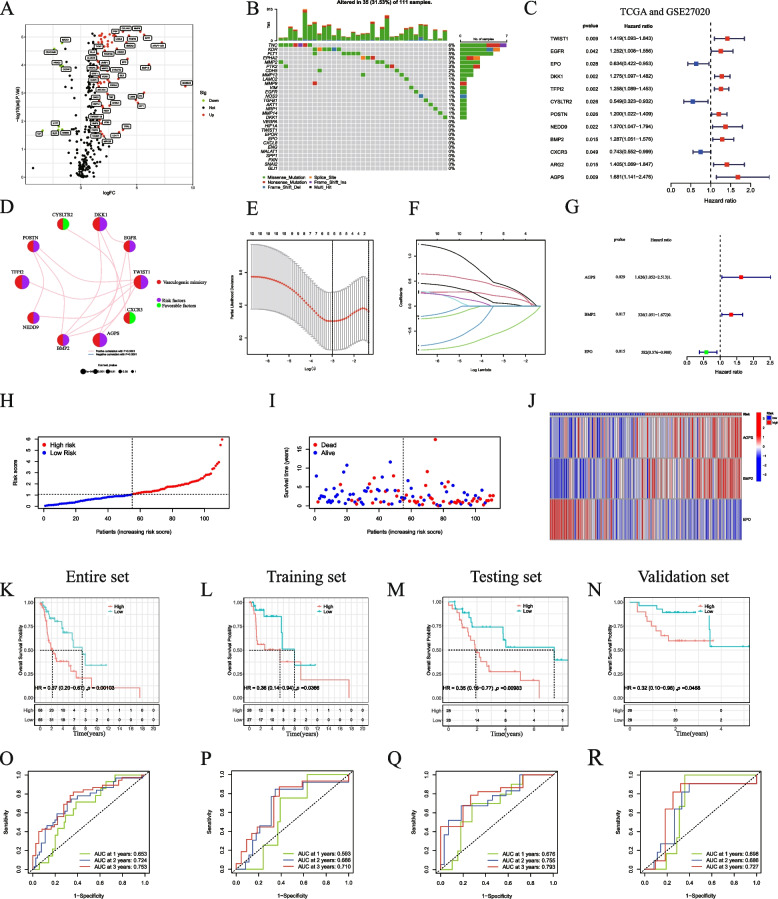
Construction of the prognostic VMRG model in LSCC. **A** Screening for VMDEGs using the TCGA database. **B** Distribution of TMB according to DEGs. **C** Twelve prognostic genes extracted from the TCGA and GEO databases using univariate Cox regression analysis. **D** Correlation analysis of prognosis-related genes. **E**–**F** A prognostic model was constructed using LASSO Cox regression. **G** Risk genes in the VMRG prognostic model. **H** Distribution of risk scores. **I** Distribution of the survival status of patients. **J** A heatmap of the three prognostic signature genes. KM survival curves and the AUC for the entire set (**K**, **O**), in the training (**L**, **P**), testing (**M**, **Q**), and validation (**N**, **R**) sets

We obtained the RNA data of 111 LSCC and 12 normal tissue samples with matching clinical information from the TCGA database. We identified a total of 334 (Table S3) VM-related genes (VMRGs) from Genecard, extracted the expression matrix of VMRGs, and screened 141 VMDEGs between normal and LSCC samples (Table S4, Fig. 1A). KEGG pathway analysis showed that VMDEGs were significantly enriched in 30 KEGG pathways, some of which were related to tumor vasculogenic mimicry and metastasis, including PI3K − Akt signaling pathway; HIF-1 signaling pathway; Focal adhesion; MAPK signaling pathway; VEGF signaling pathway (Fig. S3A-B). According to the analysis, some GO categories related to VM are enriched, including extracellular matrix structural constituent; regulation of vasculature development; regulation of angiogenesis; endothelial cell migration [28, 29] (Fig. S3C-D). Subsequently, we analyzed the mutation burden of VMDEGs (Fig. 1B). Using the TCGA and GSE27020 datasets, we performed univariate Cox regression analysis to determine the relationship between VMDEGs and LSCC prognosis (Fig. 1C). We screened 12 VMRGs, which were significantly correlated with the OS of patients with LSCC, and analyzed their correlations (Fig. 1D). Based on the 141 VMDEGs, we constructed a VMRG prognosis model that included three genes, using LASSO Cox regression analysis (Fig. 1E–G). Based on the non-significant relationship between scaled Schoenfeld residuals and time, the proportional hazards assumption was supported. There was also no pattern in the plot of scaled Schoenfeld residuals against transformed time (Fig. S4). Subsequently, we created training and testing sets (samples and clinical information are presented in Table S5) using the entire dataset. We then divided patients into high- and low-risk groups based on their median risk scores (Fig. 1H and Fig. S5A–B). Notably, we found that the number of deaths in the high-risk group was higher than that in the low-risk group (Fig. 1I and Fig. S5D–E). We then used a heatmap to visualize the expression levels of the three VMRGs in high- and low-risk patients (Fig. 1J and Fig. S5G–H). We detected that the survival probability of low-risk patients with LSCC was significantly higher than that of patients in the high-risk group in both sets (training and testing) (Fig. 1K–M). Receiver operating characteristic curve (ROC) analysis showed that the area under the curve (AUC) for 3-year OS was 0.753 in the entire set, 0.710 in the training set, and 0.793 in the internal testing set (Fig. 1O–Q). To validate the prognostic model, we calculated the risk scores for each patient in the external validation set (GSE27020) using the obtained risk score formula, and based on the median risk score, patients in the validation set were divided into high- and low-risk groups (Fig. S5C). We performed further analysis to determine the survival status of patients with LSCC in the high- and low-risk groups (Fig. S5F) and the expression distribution of the three prognostic genes (Fig. S5I). We found that consistent with the TCGA dataset, patients in the high-risk group had a poorer prognosis than those in the low-risk group in the external validation set (Fig. 1N). In addition, the AUC for 3-year OS in the external validation set was 0.727 (Fig. 1R). These data suggested that our model can accurately predict the prognosis of patients with LSCC.

### Independence of the VMRG signature in predicting OS and PFS

**Fig. 2:**
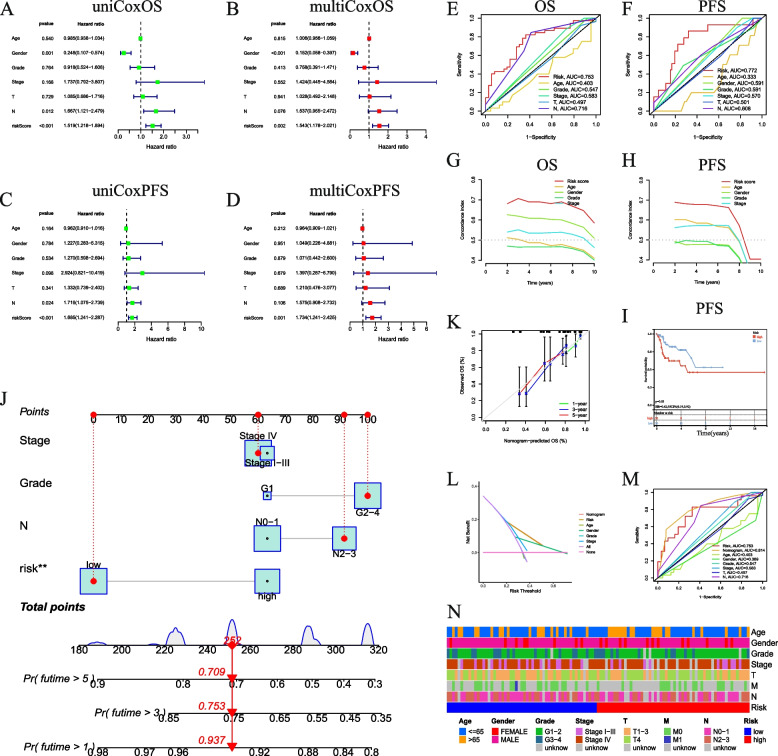
Independent prognostic analysis of the VMRG prognostic model. **A**–**D** Univariate and multivariate Cox analyses of OS and PFS in LSCC. ROC curves for OS (**E**) and PFS (**F**) according to the risk score. C-index values of the risk scores for OS (**G**) and PFS (**H**). **I** KM curves of PFS based on the risk score. **J** OS nomogram, and (**K**) calibration curve for the nomogram; (**L**) ROC analysis of the nomogram; (**N**) Histogram statistics of clinical features

**Fig. 3:**
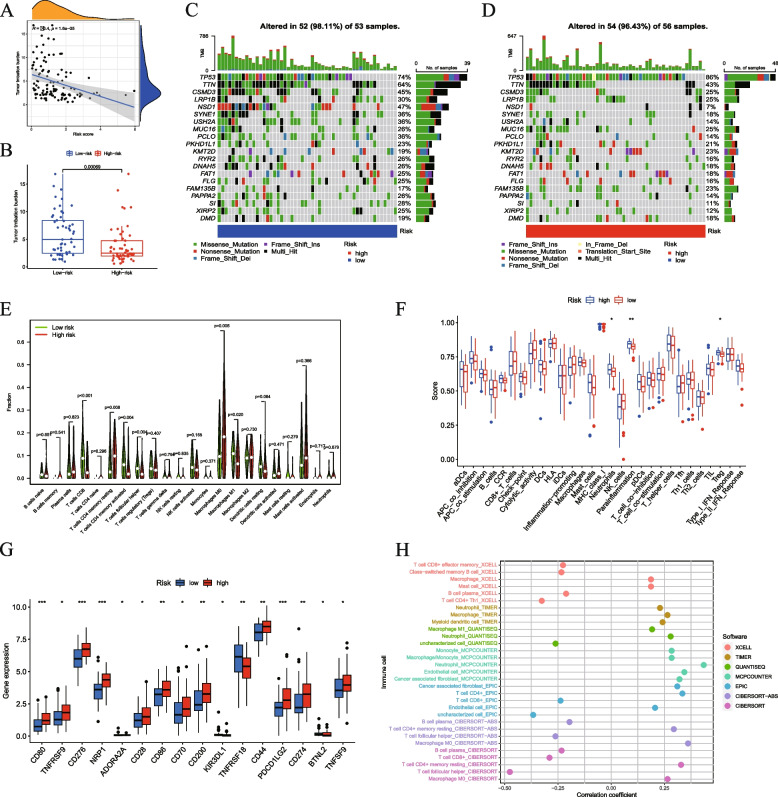
TMB and TME infiltration. **A** Relationship between TMB and risk score. **B** Comparison of TMB scores between risk groups. **C** TMB analysis of high- and low-risk groups. **D** CIBERSORT analysis of immune cell infiltration in high- and low-risk groups. **E** ssGSEA analysis in high- and low-risk groups. **F** Comparison of immune check loci in high- and low-risk groups. **G** Relationship of the risk score and abundance of immune cells in LSCC

To further explore the prognostic value of the VMRG signature, we performed univariate and multivariate Cox regression analyses. We found that this signature had independent prognostic significance for the OS (Fig. 2A, B) and PFS (Fig. 3C, D) of patients with LSCC. In addition, the AUC for 3-year OS and 3-year PFS was 0.753 (Fig. 2E) and 0.772 (Fig. 2F), respectively, which were significantly higher than those of other clinical parameters. According to the C-index and DCA, the risk model predicted the prognosis of LSCC better than other clinical factors (Fig. 2G–H). Moreover, the difference in PFS between the high- and low-risk patients was statistically significant (*P* = 0.006; Fig. 2I). We assessed the survival rate based on a nomogram created using the risk-score and various clinical indicators (Fig. 2J). We first performed calibration analysis to determine the discriminative ability and clinical utility of the nomogram. We observed that the predicted OS from the nomogram was highly similar to the observed OS (Fig. 2K). For predictive accuracy, both ROC and decision curve analyses (DCA) showed that the nomogram had a higher predictive capability and accuracy than any single clinical feature, highlighting it as beneficial for clinical decision-making and treatment planning (Fig. 2L–M). We further stratified patients based on their clinical features as demonstrated in a histogram (Fig. 2N).

### TMB analysis, functional enrichment, and immune cell infiltration of DEGs

We performed Gene Ontology (GO) and Kyoto Encyclopedia of Genes and Genomes (KEGG) analyses to assess the enrichment of 440 DEGs between the two risk groups (Table S6). KEGG pathway analysis indicated that these genes were mainly associated with focal adhesion, the PI3K-Akt signaling pathway, and the calcium signaling pathway (Fig. S6A, B). GO analysis showed associations between the extracellular matrix, wound healing, and signaling receptor activation (Fig. S6C, D). We performed Gene Set Enrichment Analysis (GSEA) to assess potential pathways in the two groups. We found that the high-risk group was primarily enriched in cytokine-cytokine receptor and ECM-receptor interactions, whereas the low-risk group was primarily enriched in allograft rejection (Fig. S6E, F). Subsequently, TMB analysis showed that the TMB score was negatively correlated with the risk score (Fig. 3A) and was lower in the high-risk group (Fig. 3B). We then identified 20 significant gene mutations in both groups and determined their mutation status (Fig. 3C and D).

Using the CIBERSORT algorithm, we analyzed the levels of immune cell infiltration in the high- and low-risk groups. We determined that resting memory CD4 + T-cells and M0 macrophages were highly expressed in the high-risk group. In contrast, CD8 + T-cells, activated memory CD4 + T-cells, and M1 macrophages were abundant in the low-risk group (Fig. 3E). We further analyzed the infiltration abundance of immune cells and their correlations in patient samples (Fig. S7A, B). Using the ssGSEA algorithm, we examined the distribution of immune cells in the two groups. Parainflammatory cells, neutrophils, and tumor-infiltrating lymphocytes (TILs) were more abundant in the high-risk group (Fig. 3F). Moreover, we noticed that several effective checkpoint immunotherapy targets, such as CD80, CD86, CD276, and CD274 were highly expressed in the high-risk group (Fig. 3G). We also used multiple platforms (TIMER, CIBERSORT, CIBERSORT-ABS, QUANTISEQ, XCELL, MCPCOUNTER and EPIC) to study the correlation between risk scores and immune cell types. As shown in the bubble chart in Fig. 3, M0 macrophages, neutrophils, and endothelial cells were positively correlated with the risk score, whereas CD8 + T-cells, plasma B-cells, and follicular helper T-cells were negatively correlated with the risk score (Fig. 3H). Thus, immune cell infiltration may affect patient outcomes, further providing insights into targeted immunotherapy for LSCC.

### Clustering analysis of VMRGs in LSCC

**Fig. 4:**
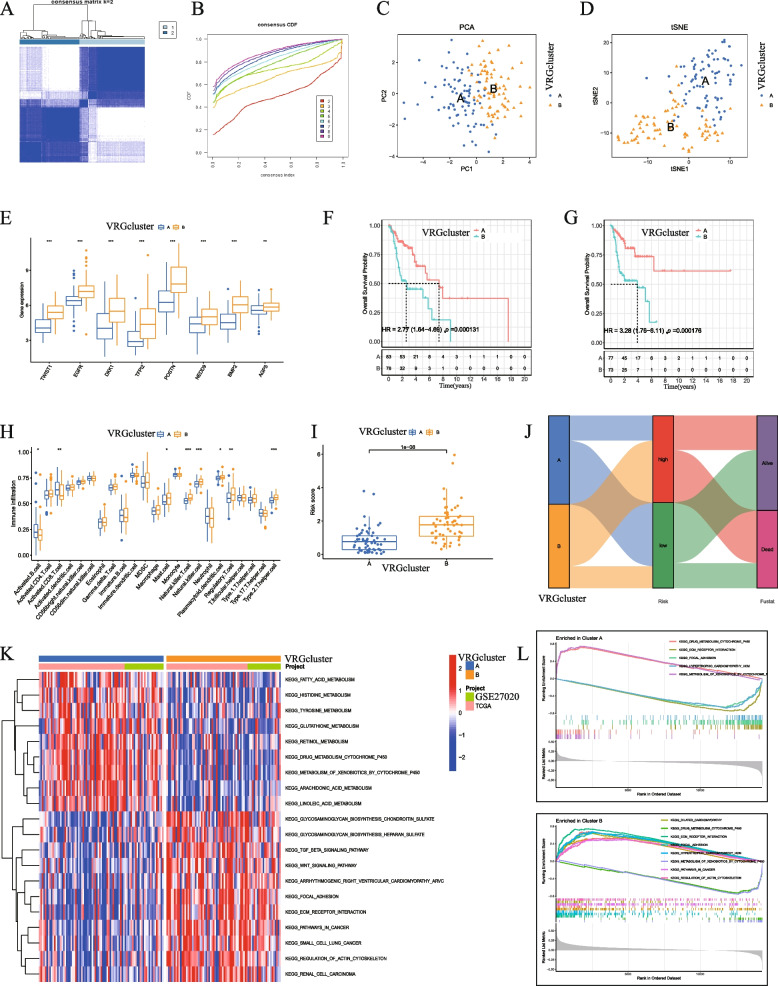
Clustering analysis of VMRGs in LSCC.** A**–**B** LSCC samples (TCGA and GSE27020) were divided into 2 clusters using the consensus clustering method. **C** PCA analysis of cluster 1 and cluster 2. **D** t-SNE analysis of clusters 1 and 2. **E** Differential expression of prognostic genes in clusters 1 and 2. **F**–**G** The KM plot of the two clusters of patients with LSCC for determination of OS and PFS. **H** Differences in immune cell infiltration between the two clusters using ssGSEA. **I**–**J** The distribution of risk scores in the two clusters. **K**–**L** GVSA and GSEA enrichment analysis of differential genes between the two clusters

We used a consensus clustering method to study the molecular subtypes of LSCC based on the VMDEGs (TCGA and GSE27020) related to OS outcomes. Using consensus clustering, we divided patients with LSCC into two subtypes (cluster 1, *n* = 83; cluster 2, *n* = 76; Table S7), with K = 2 as the optimal value (Fig. 4A, B). We used principal component analysis (PCA) and tSNE to separate the two clusters according to their distribution characteristics (Fig. 4C, D). We then analyzed the differential expression of OS-related genes in the two clusters and found that TWIST1, EGFR, DKK1, TFPI2, POSTN, NEDD9, BMP2, and AGPS were highly expressed in Group B (Fig. 4E). To determine the stability and reliability of molecular subtypes, we generated KM curves to confirm the differences in the survival rate between the two subtypes. We identified that, compared with cluster 1, the survival rate (both OS and PFS) of patients in cluster 2 was worse (*P* < 0.05, Fig. 4F, G). Using the ssGSEA algorithm, we calculated and displayed the distribution of infiltrating immune cells in the two clusters. We observed that natural killer (NK) and type 2 T-cells (T-helper cells) were highly expressed in cluster 2, whereas activated CD8 T-cells and activated B-cells were highly expressed in cluster 1 (Fig. 4H). We detected a significant difference in the risk scores between the two clusters (Fig. 4I). In particular, the cluster with a higher risk score had a poorer prognosis, suggesting that samples in cluster 2 mainly belonged to the high-risk patient group (Fig. 4J). GSVA analysis revealed the differential expression of KEGG pathways between the two groups, with the TGF-β and Wnt signaling pathways being highly enriched in cluster 2 (Fig. 4K). Likewise, GSEA indicated that drug metabolism, extracellular matrix (ECM)-receptor interaction, and focal adhesion were highly enriched in cluster 2 (Fig. 4L).

### Screening of core prognostic genes in LSCC

**Fig. 5:**
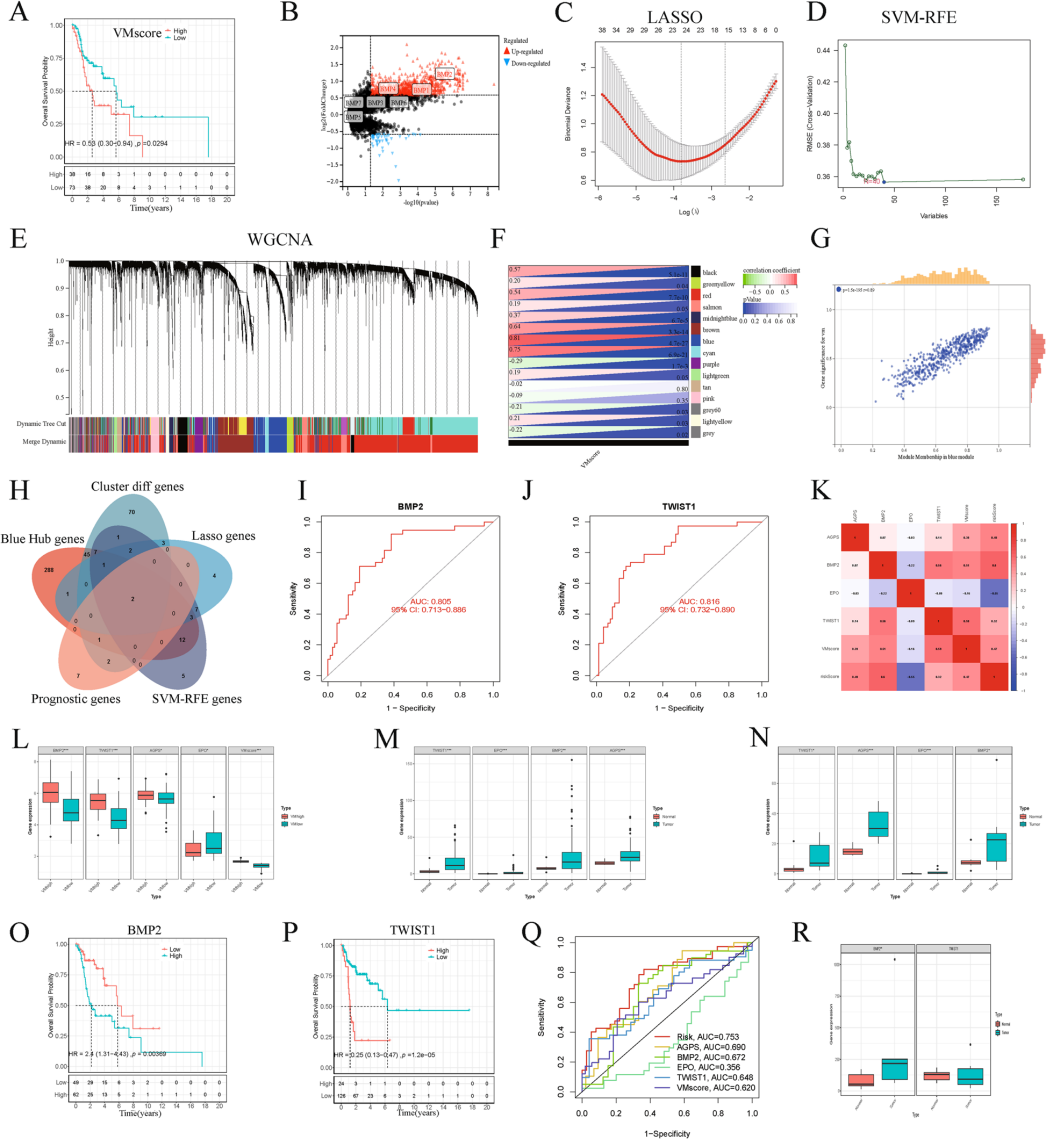
*BMP2* as the core prognostic VMRG for LSCC progression. **A** KM curve based on VM scores in LSCC. **B** Differentially expressed genes in high-and low-VM score groups. **C**–**D** LASSO and SVM-RFE screening of candidate diagnostic genes. **E** WGCNA of differentially expressed genes. **F** Correlations of VM scores with WGCNA modules. **G** Correlation between the blue module and VM scores. **H** Five algorithmic Venn diagram screening genes. **I**–**J** The AUC of *BMP2* and *TWIST1*. **K** Heatmap of gene correlation analysis. **L** Differences in the expression of two core genes between high- and low-VM score groups. **M**–**N** Differential expression of two core genes between normal and tumor samples. **O**–**P** KM survival analysis of *BMP2* and *TWIST1* in LSCC. **Q** ROC curves of survival. **R** Differential expression of two core genes in GSE51985 data

To elucidate the potential mechanisms of LSCC malignant progression, we explored the relationship between VMRGs and the malignant progression of LSCC. Using ssGSEA, we calculated VM scores based on 334 VMRGs (Table S8). KM analysis revealed that patients with higher VM scores had worse OS (Fig. 5A). Based on VM scores, patients were divided into high- and low-VM score groups, and 134 differentially expressed genes were obtained between the two groups (Fig. 5B). To select and validate the feature genes, we performed LASSO regression analysis on the high- and low-VM score groups and obtained 23 genes (Fig. 5C). We further used the SVM-RFE algorithm and obtained an additional 40 genes (Fig. 5D). Subsequently, to determine which specific genes might be related to VM, we performed weighted correlation network analysis (WGCNA) on the LSCC samples [30]. We identified 15 gene modules (Fig. 5E), with the blue module showing the highest correlation with the VM scores (Fig. 5F, G). We selected a total of 360 core genes from the blue module. Using the above algorithms, we sought to determine the intersection of genes and identified *BMP2* and *TWIST1* (Fig. 5H and Table S9). We used the ROC curve to measure the ability to discriminate between patients with high and low VM scores. We found that the AUCs for *BMP2* and *TWIST1* were 0.806 and 0.816, respectively (Fig. 5I, J). Correlation analysis showed that *BMP2* and *TWIST1* were positively correlated with the VM and risk scores (Fig. 5K). In addition, *BMP2* and *TWIST1* were highly expressed in the high-VM score group (Fig. 5L). According to their expression in TCGA patients, both unpaired and paired analyses indicated that *BMP2* and *TWIST1* were highly expressed in tumor samples (Fig. 5M, N). KM analysis indicated that patients with high expression of *BMP2*, *TWIST1*, and *AGPS* had significantly lower survival rates than those with low expression of these genes, whereas the opposite was true for *EPO* (Fig. 5O, P and Fig. S8A, B). Survival ROC analysis revealed that the AUCs for *BMP2* and *TWIST1* were 0.672 and 0.648, respectively (Fig. 5Q). Furthermore, based on the GSE51985 data, we found that *BMP2* was highly expressed in tumor tissues compared with normal tissues; however, no significant difference was observed for the expression of *TWIST1* (Fig. 5R). Consequently, as one of the prognostic genes of the VMRG signature, we subsequently focused on exploring the specific biological function of *BMP2* as a core gene in the malignant progression of LSCC.

### Expression of *BMP2* in LSCC and its relationship with clinical features

**Fig. 6:**
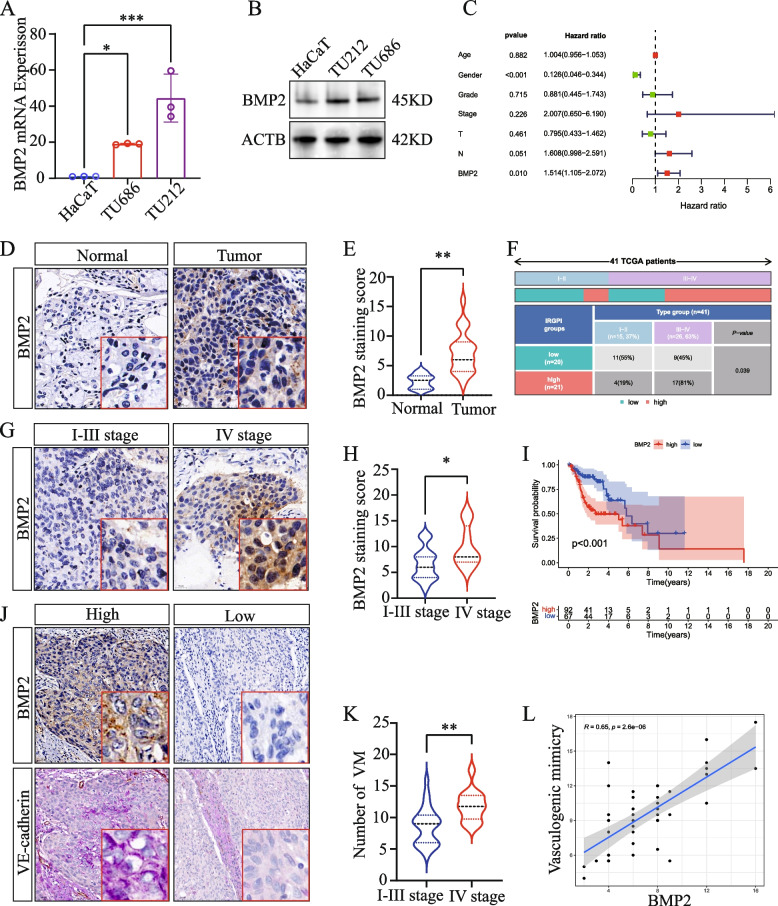
The expression of *BMP2* in LSCC and its relationship with clinical features. **A** Expression analysis of *BMP2* using qRT-PCR. **B** WB analysis of the expression of *BMP2*. **C** Multivariate Cox regression analyses. **D**, **G** Representative figures of IHC staining for *BMP2*. **E**, **H** Statistical analysis of IHC staining intensity. **F** Histogram of chi-square test. **I** KM survival curve of *BMP2* on OS in TCGA and GEO cohorts. **J** VM vessels (CD3-/PAS +) and *BMP2* were observed by staining LSCC. **K** Statistical analysis of the number of VM. **L** The analysis of the correlation between the number of VM and *BMP2*. *P < 0.05, **P < 0.01, ***P < 0.001, Student’s *t*-test and one-way ANOVA

Subsequent qRT-PCR and WB analyses indicated that *BMP2* was highly expressed in LSCC cell lines (Fig. 6A, B). Multivariate analysis of the four genes showed that in LSCC, *BMP2*, and *TWIST1* were independent risk factors related to OS (Fig. 6C and Fig. S9A), whereas *EPO* acted as a protective factor (Fig. S9B), and *AGPS* was not significantly associated with OS (Fig. S9C). IHC analysis of LSCC tissues (HN049La01) showed that *BMP2* was highly expressed in LSCC tissues compared with that innormal laryngeal mucosal tissues (Fig. 6D, E). Moreover, we detected that its expression in stage IV LSCC tissues was significantly higher than that in stages I–III (Fig. 6G, H). The chi-square test results indicated that a higher proportion of patients with stage III–IV disease exhibited high expression of *BMP2* compared with patients at other stages (Fig. 6F). According to the TCGA and GEO data, the survival rate of patients with high expression of *BMP2* was lower than that in patients in the low-expression group (Fig. 6I). Additionally, IHC analysis showed that BMP2 expression in LSCC tissues is positively correlated with the number of VMs (Fig. 6J, L), and the number of VMs is significantly higher in stage IV patients compared to stages I-III (Fig. 6K).

### In vitro knockdown of *BMP2* inhibited LSCC VM and metastasis

**Fig. 7:**
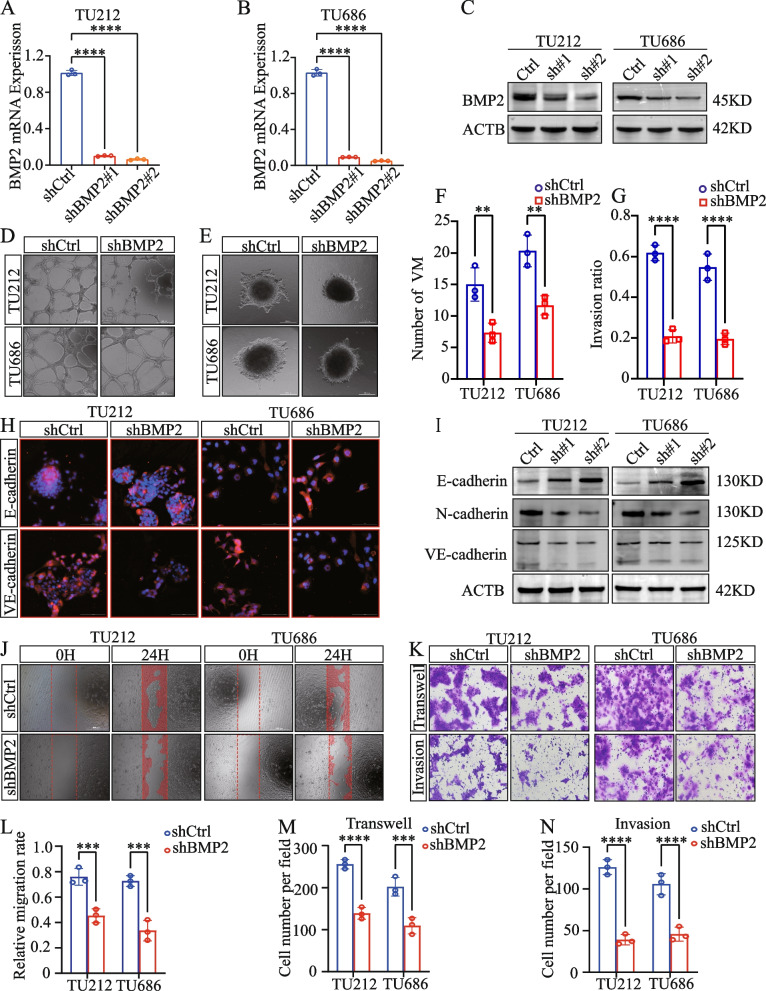
In vitro knockdown of *BMP2* inhibits LSCC VM and metastasis. **A**-**C** Validation of *BMP2* knockdown by qRT-PCR and western blotting. **D** Tubule formation assay of TU212 and TU686 cells. **E** 3D cell spheroid invasion assay of TU212 and TU686 cells. **F**–**G** Column graph in index of tubule formation and spheroid invasion ratio. **H** Immunofluorescence (IF) revealed that changes in the expression of *BMP2* altered the expression of EMT markers. **I** Western blot analysis of EMT markers. Scratch wound-healing (**J**) and Transwell migration and invasion (**K**) assays performed in TU212 and TU686 cells. ***P < 0.001, ****P < 0.0001, Student’s *t*-test and two-way ANOVA

To verify the role of BMP2 in LSCC VM and metastasis, we first knocked down the expression of *BMP2* in LSCC cell lines and confirmed the knockdown efficiency using qRT-PCR and WB analyses (Fig. 7A–C). Subsequent tube formation assays revealed that *BMP2* knockdown inhibited LSCC VM (Fig. 7D, F). A sprouting invasion assay indicated that *BMP2* knockdown significantly suppressed the sprouting rate in LSCC (Fig. 7E, G). Moreover, cellular immunofluorescence experiments revealed that upon *BMP2* knockdown, the fluorescence intensity of E-cadherin in LSCC cells was increased, whereas that of VE-cadherin was decreased (Fig. 7H). Likewise, WB analysis indicated that following the suppression of BMP2, the protein levels of E-cadherin were increased, whereas those of VE-cadherin and N-cadherin were decreased (Fig. 7I). These findings suggested that BMP2 promotes epithelial-mesenchymal transition (EMT) in LSCC. Furthermore, scratch assays revealed that *BMP2* knockdown inhibited the healing ability of LSCC cells (Fig. 7J, L). Finally, Transwell assays confirmed that *BMP2* knockdown inhibited the metastasis and invasion capabilities of LSCC cells in vitro (Fig. 7K, M, N).

### In vivo knockdown of *BMP2* inhibits LSCC VM and metastasis

**Fig. 8:**
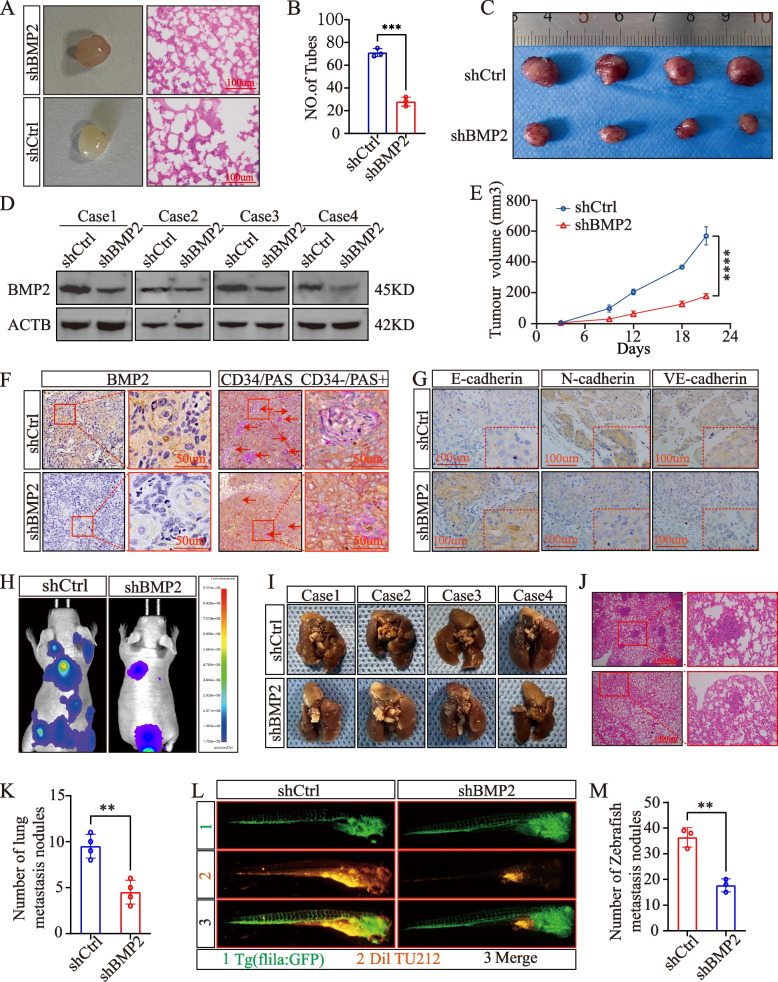
In vivo knockdown of *BMP2* inhibits LSCC VM and metastasis. Representative Matrigel plugs are shown, *n* = 3. **B** ImageJ software was used to count and analyze tubules. **C** Images of solid tumors from nude mice. **D** Curves of xenograft tumor growth in nude BALB/c mice (*n* = 4). **E** Western blot analysis of the expression of BMP2 in xenograft tumors. **F** IHC staining for *BMP2* and CD34/PAS, CD34 − PAS + vasculogenic mimicry (VM) tubes. **G** IHC analysis of EMT markers in TU212 xenografts. **H**–**J**) Lung metastasis model. **K** Statistical analysis of nodules. **L**, **M** A zebrafish model was used to analyze the dissemination and metastasis of TU212 cells. **P < 0.01, ***P < 0.001, Student’s *t*-test

We also conducted a series of in vivo experiments. Matrigel plug assays in nude mice confirmed that *BMP2* knockdown inhibited VM in LSCC (Fig. 8A, B). In addition, subcutaneous tumorigenesis experiments in nude mice showed that *BMP2* knockdown inhibited LSCC growth (Fig. 8C, E). We found that the level of protein expression of BMP2 in the tumor tissues of mice in the knockdown group was lower than that in tissues of mice in the normal group, as indicated by WB (Fig. 8D). CD34/PAS staining showed that after *BMP2* knockdown, the number of formed VMs within the tumor was significantly decreased (Fig. 8F). Furthermore, IHC indicated that after *BMP2* knockdown, the expression of E-cadherin was upregulated, whereas the protein levels of VE-cadherin and N-cadherin were downregulated (Fig. 8G). We also observed that the number of metastatic lung nodules in a lung metastasis model in nude mice was significantly lower after *BMP2* knockdown than that in the control group (Fig. 8H–K). Additionally, after *BMP2* knockdown, the number of metastatic clusters in zebrafish was significantly lower than that in the control group (Fig. 8I–M).

### BMP2 promoted LSCC VM and metastasis by activating the PI3K-AKT pathway

**Fig. 9:**
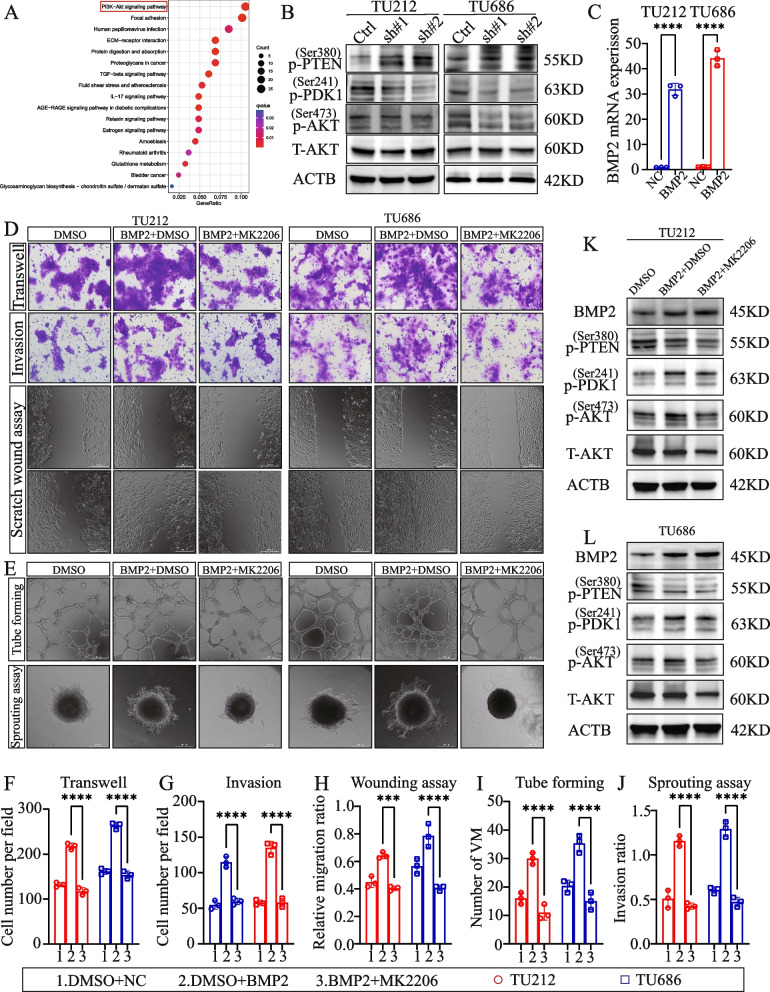
*BMP2* promotes LSCC VM and metastasis by activating the PI3K-AKT pathway. **A** KEGG enrichment analysis of differentially expressed genes. **B** Western blot analysis of p-PTEN, p-PDK, p-Akt, and Akt in LSCC. **C** Overexpression efficiency of BMP2 was verified using qRT-PCR. **D**, **F**–**H** Transwell migration, Transwell invasion, and scratch wound-healing assays were performed in three groups (DMSO, BMP2 + DMSO, and BMP2 + MK2206), respectively. **E**, **I**, **J** Tubule formation and spheroid invasion assays were conducted in the same three groups. **K**, **L** Western blot analysis of *BMP2*, p-PTEN, p-PDK, p-Akt, and Akt in the three groups in TU212 and TU686 cells. ***P < 0.001, ****P < 0.0001, Student’s *t*-test and two-way ANOVA

Using the TCGA and GEO datasets, we identified DEGs between patients with high and low expression of *BMP2*. KEGG enrichment analysis revealed that these DEGs were significantly enriched in the PI3K-AKT signaling pathway and focal adhesion (Fig. 9A). We used the GSVA package in R software to analyze the head and neck squamous cell carcinoma (HNSCC) data from TCGA, and found that the expression of *BMP2* was positively correlated with the PI3K-AKT pathway, angiogenesis, and EMT-markers (Fig. S10A, B, C). Subsequently, we noticed that the downregulation of BMP2 resulted in increased levels of p-PTEN, whereas the levels of p-PDK1 and p-AKT were decreased, as indicated by WB (Fig. 9B). We then used qRT-PCR to confirm the overexpression efficiency of *BMP2* (Fig. 9C). Transwell and scratch assays showed that AKT inhibition reversed the ability of BMP2 to promote LSCC metastasis and invasion (Fig. 9D, F, G, H). Further experiments demonstrated that overexpressing BMP2 enhanced tube formation in LSCC and the sprouting rate of LSCC cells; however, MK2206, the Akt inhibitor, weakened this effect (Fig. 9E, I, J). We also observed changes in the phosphorylation levels of the PI3K-AKT signaling pathway in LSCC cells after overexpressing BMP2, with or without the addition of MK2206 (Fig. 9K, L).

### BMP2 upregulation in LSCC resulted from increased transactivation by RUNX1

**Fig. 10:**
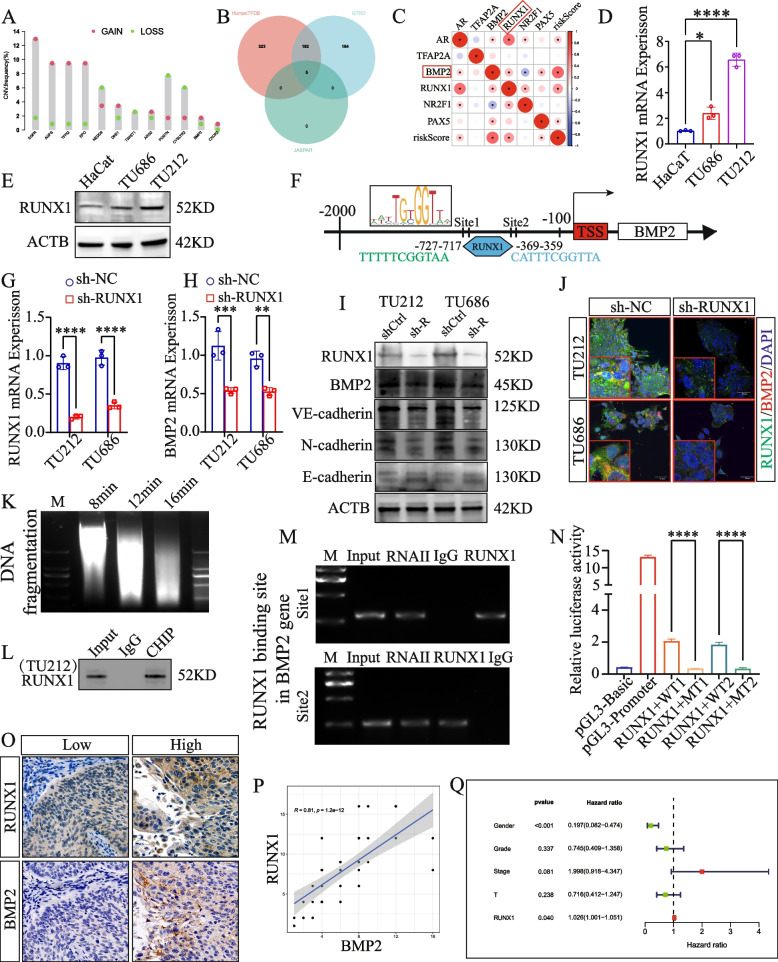
*BMP2* upregulation in LSCC results from increased transactivation by RUNX1. **A** CNV analysis of 12 prognostic VMRGs. **B** Venn diagram of predicted transcription factors. **C** Heatmap of correlation analysis. **D**, **E** QRT-PCR and western blotting shows the overexpression of *RUNX1* in LSCC cells. **F** Diagram of *RUNX1* binding sites. **G** qRT-PCR of the expression of *RUNX1* and *BMP2*. **I** Western blot analysis of *BMP2*. **J** Immunofluorescence assay, *RUNX1* (red fluorescence); *BMP2* (green fluorescence). **K** Upon shearing by sonication, chromatin fragments ranged from 100 to 500 bp in size. **L** Western blotting was used to detect the *RUNX1* in the ChIP assay. **M** DNA-binding sites electrophoresed on agarose gels. **N** Dual-luciferase reporter assays were used to analyze luciferase activity. **O**–**P** The levels of expression of *BMP2* and *RUNX1* were correlated according to IHC staining. **Q** Multivariate analysis of *RUNX1* **P < 0.01, ***P < 0.001, ****P < 0.0001, one-way ANOVA and two-way ANOVA

According to the TCGA data, the copy number variation (CNV) rate of the prognostic gene *BMP2* was only slightly altered in LSCC (Fig. 10A). Therefore, we investigated whether the upregulation of *BMP2* was due to transcriptional activation by transcription factors upstream of *BMP2*. By predicting the transcription factors related to *BMP2* using the AnimalTFDB, GTRD, and JASPAR databases and determining their intersection (Fig. 10B and Table S10), we identified five transcription factors (TFAP2A, RUNX1, PAX5, AR, and NR2F1). We then analyzed the correlation between these five transcription factors, risk score, and expression of *BMP2*. We detected that the expression of RUNX1 was significantly positively correlated with the risk score and expression of *BMP2* (Fig. 10C). We investigated the expression of RUNX1 in LSCC. We performed both qRT-PCR and WB and found a high expression of RUNX1 in LSCC cell lines (TU212 and TU686) compared with that in HaCaT cells (Fig. 10D, E). In addition, staining of laryngeal cancer tissue microarrays revealed an overexpression of RUNX1 in patient tissues, with a notably higher expression in stage IV patients than that in patients at stages I–III (Fig. S11). Using the prediction website JASPAR, we performed analysis of the *BMP2* promoter sequence and found that RUNX1 might bind to *BMP2* at positions -369 to -359 and -727 to -717 (Fig. 10F). We also observed that after *RUNX1* knockdown in an LSCC cell line, the expression of *BMP2* was significantly downregulated, and the EMT process was inhibited (Fig. 10G-J). Our ChIP agarose gel electrophoresis assay also provided evidence that RUNX1 directly interacts with the predicted binding sites on the *BMP2* promoter, leading to the reactivation of *BMP2* (Fig. 10K-M). This finding was further supported by a luciferase reporter assay (Fig. 10N). To investigate the relationship between *RUNX1* and the expression of *BMP2* in LSCC tissues, we performed IHC, which revealed a positive correlation between the expression levels of *RUNX1* and *BMP2* (Fig. 10O, P). Furthermore, multivariate regression analysis of samples from the TCGA database revealed *RUNX1* to be an autonomous risk factor for unfavorable prognosis in LSCC (Fig. 10Q). Based on these results, we concluded that the overexpression of *BMP2* in LSCC was attributed to its increased transactivation by *RUNX1*.

### RUNX1 regulates BMP2 to promote vasculogenic mimicry in LSCC

**Fig. 11:**
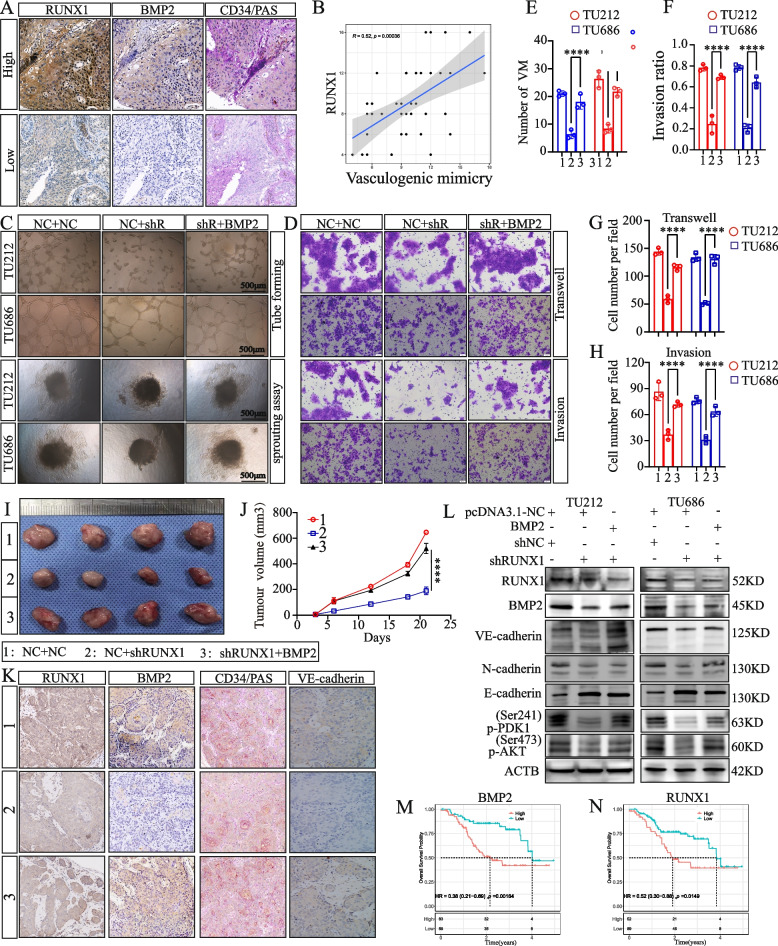
RUNX1 regulates BMP2 to promote vasculogenic mimicry in LSCC. **A**-**B** IHC results of *RUNX1, BMP2* and CD34/PAS in LSCC tissue. **C**, **E**, **F** Tubule formation and spheroid invasion assays. **D**, **G**, **H** Transwell migration assay and transwell invasion assay. **I** Three groups (NC + NC, NC + shRUNX1, shRUNX1 + BMP2) of solid tumors were observed. **J** Curves of xenograft tumor growth in nude BALB/c mice (*n* = 4). **K** IHC staining for RUNX1, BMP2 and CD34/PAS in tumor tissues of nude mice. **L** Western blot analysis of EMT-related proteins and the AKT signaling pathway. **M**, **N** Kaplan–Meier curve for the 5-year overall survival rate of *RUNX1* and *BMP2*. **P < 0.01, ***P < 0.001, ****P < 0.0001, one-way ANOVA and two-way ANOVA

The staining of laryngeal cancer tissue microarrays shows that the expression of *RUNX1* in patient tissues is positively correlated with the number of VMs (Fig. 11A, B). The in vitro experiments, including tubule formation, spheroid invasion and transwell assays, provided evidence supporting the notion that *RUNX1* regulates *BMP2* to promote vasculogenic mimicry in LSCC (Fig. 11C-H). Subsequently, the subcutaneous tumor formation in nude mice also confirmed that *BMP2* can reverse the growth inhibition caused by *RUNX1* knockdown, and also reverse the reduction in vasculogenic mimicry density (Fig. 11I-K). Furthermore, We found that upon suppressing *RUNX1*, the activity of the PI3K-AKT signaling pathway was reduced in LSCC cells, consequently inhibiting EMT. Notably, this phenomenon was reversed by overexpressing *BMP2* (Fig. 11L). Finally, through KM analyses of 5-year OS, we observed that patients with LSCC with high expression of *RUNX1* and *BMP2* exhibited a significantly worse 5-year OS rate (Fig. 11M, N).

## Discussion

Vasculogenic mimicry (VM) is a recently identified angiogenic mechanism observed in numerous malignant tumors, which differs from conventional angiogenic processes mediated by the vascular endothelium. VM entails the development of microvascular channels comprised of tumor cells, facilitating the provision of blood supply for tumor proliferation, thus representing a novel neovascularization model in invasive tumors [4, 31]. A growing body of evidence has suggested that VM correlates with a poorer overall survival rate in patients with malignant tumors and may, therefore, be a promising therapeutic target [32, 33]. Strategies to disrupt VM include the use of antiangiogenic drugs, drugs targeting EMT, and cancer stem cell (CSC) treatments. VM channels have been reported to be reduced in HNSCC following *EPHA2* knockdown in vitro, with the expression of *EPHA2* being positively correlated with that of EMT-related markers, such as TWIST and F-actin [34]. The role of VMRGs in LSCC remains unclear. Therefore, we constructed a prognostic model using VMRGs to predict the prognosis of patients with LSCC. Here, we demonstrated the existence of an important regulatory axis in the tumor microenvironment involving the upregulation of BMP2 by RUNX1, which in turn activates the PI3K-AKT signaling pathway, subsequently promoting EMT and VM, both of which ultimately promote the malignant progression of LSCC.

In recent years, LSCC biomarkers, prognostic markers, and prognostic models have received increasing attention [35, 36]. Patients with LSCC may benefit from these models given their ability to predict their prognosis. The prognostic model constructed in this study accurately predicted the prognosis of patients with LSCC in line with the findings of previous studies.

Several studies have suggested that TMB is an immunotherapy biomarker, and patients with high TMB scores are more likely to benefit from immunotherapy [37]. However, our results indicated that the TMB score was lower in patients in the high-risk group, making it particularly important to seek effective immunotherapeutic approaches for LSCC. Cancer progression is associated with the disruption of the immune microenvironment, which promotes tumor invasion and metastasis [38]. Some studies have suggested that LSCC treatments should be actively combined with immunotherapy. Increasing evidence support that T-cells play an important role in immunotherapy. CD8 + T-cells have been associated with better outcomes in esophageal, colorectal, and nonsmall cell lung cancers [39, 40]. Our data showed that the expression of CD8 + T-cells in the high-risk subgroup was lower than that in the low-risk subgroup, in consistency with the results of previous studies. Tumor-associated macrophages (TAMs), which have two functional states, M1 and M2, are essential components of the tumor immune microenvironment. M0 macrophages can transform into M1 or M2 macrophages; M1 macrophages mainly promote antitumor immunity by killing pathogens and tumor cells, whereas M2 macrophages promote tumor progression [41]. Our study showed that patients in the high-risk group had a high abundance of M0 macrophages, whereas the number of M1 macrophages was notably lower than that in the low-risk group. Overexpression of CD274 in certain cancers contributes to tumor progression and helps evade immune surveillance [42]. Accordingly, we found that CD276 and CD274 were highly expressed in the high-risk group.

Using the SVM-RFE algorithm and WGCNA, we identified core prognostic genes, among which *BMP2* was one of the most crucial. In nasopharyngeal carcinoma, the overexpression of *BMP2* has been reported to enhance tumor cell invasion and EMT through the mTORC1 signaling pathway [43]. Studies have shown that BMP2 is a secretory protein highly expressed in various cancers that facilitate cell invasion, metastasis, and EMT [44]. However, its clinical significance and biological functions in LSCC remain unclear. We observed that patients with high expression of BMP2 in the combined TCGA and GEO dataset had shorter OS than those with low expression; after adjusting for other factors in multivariate analysis, this association was demonstrated to be independent. To explore the mechanism underlying the effects of BMP2 on LSCC progression, we performed functional experiments, which revealed that inhibiting the expression of BMP2 suppressed the VM and migration of LSCC cells.

KEGG analysis also indicated that the PI3K-AKT signaling pathway was highly enriched in patients with LSCC with high and low expression of BMP2. The PI3K/AKT/mTOR signaling pathway is one of the most activated signaling pathways in cancer, promoting tumor development [45]. Activation of this signaling pathway is primarily caused by functional deficiencies in PTEN, TSC1, and TSC2 [46]. Here, we found that overexpression of *BMP2* induced the activity of PI3K-AKT, whereas inhibited that of p-PTEN in LSCC cells. Moreover, TCGA database analysis revealed that the expression of BMP2 was positively correlated with that of EMT markers in HNSCC samples. These results indicated that BMP2 promotes VM and metastasis in LSCC by activating the PI3K-AKT signaling pathway. Currently, there is a lack of selective and effective inhibitors of BMP2; hence, targeting PI3K-AKT may be a new strategy to halt LSCC progression.

To investigate the mechanism by which the expression of BMP2 is upregulated in LSCC, we investigated whether the upregulation of BMP2 was caused by transcriptional activation. Previous studies have reported that SOX9 directly binds to the promoter region of *BMP2* and increases its expression. Based on the promoter sequence of *BMP2*, JASPAR predicted that RUNX1 may be a regulatory transcription factor of *BMP2*. The regulation of cancer metastasis, angiogenesis, tumor stem cell properties, and resistance to anticancer drugs involves both indirect and direct biological functions of RUNX1 [47]. In colorectal cancer, the activation of the lncRNA RNCR3/AKT signaling axis by RUNX1 promoted tumor invasion [48]. Furthermore, as a prognostic biomarker for OS [49], RUNX1 was found to be highly expressed in HNSCC. Conversely, loss of this gene alleviated cell migration and invasion in HNSCC [50]. To our knowledge, for the first time, we discovered that RUNX1 positively regulated the expression of *BMP2* in LSCC. Moreover, ChIP-PCR results confirmed that *BMP2* is a RUNX1-regulated target gene and further verified the detailed binding site of RUNX1 in the *BMP2* promoter. Therefore, elucidating the mechanism by which RUNX1 activates the expression of BMP2 is of great significance for achieving precision therapy in LSCC.

As our understanding of VM deepens, VM holds increasing promise as a therapeutic target and a prognostic indicator. Continued research into the intricacies of VM and its clinical implications is essential for advancing our ability to combat this challenging aspect of cancer progression. This study revealed the role and detailed mechanism of BMP2 in VM and metastasis in LSCC. Based on our findings, quantification of BMP2 can help predict recurrence and survival rates before treatment. In conclusion, our findings offer new insights into the clinical application of VM in LSCC and provide new opportunities for treating LSCC resistant to antiangiogenic treatments. However, our study had certain limitations. First, the source of our tumor samples may not be representative of a broader population. Second, genetic, environmental, and lifestyle differences affect tumor behavior; therefore, the applicability of BMP2 as a biomarker in different populations needs to be further validated. Although we verified that the PI3K-AKT signaling pathway is a mediator of the function of BMP2, BMP2 may act through other pathways that were not explored in this study.

## Conclusion

In this study, a prognostic model was developed using the expression of BMP2, APS, and EPO to effectively predict the prognosis of patients with LSCC. Our findings indicated that these genes have the potential to serve as biomarkers for predicting overall survival in patients with LSCC. Furthermore, the downstream signaling pathway of BMP2 was elucidated through qRT-PCR and immunoblotting analyses, while the upstream regulatory transcription factors regulating the expression of BMP2 were explored using ChIP and luciferase reporter assays. To summarize, the upregulation of BMP2 in LSCC plays a significant role in promoting VM and metastasis, while also exhibiting a negative correlation with the prognosis of patients with LSCC. This overexpression of BMP2 in LSCC was mechanistically attributed to enhanced reverse transcription activation by RUNX1, subsequently activating the PI3K/AKT signaling pathway.

### Supplementary Information

**Additional file 1: Supplementary Fig. 1.** STR identification certificates of the three cell lines involved in this study, hacat, tu212, and tu686.

**Additional file 2: Supplementary Fig. 2.** Three cells were free of mycoplasma contamination, as determined by MycoProbe mycoplasma detection kit.

**Additional file 3: Table S1.** The sequence of the *BMP2* promoter region.

**Additional file 4: Table S2.** Wild-type and mutant sequences of binding sites in the *BMP2* promoter region.

**Additional file 5: Table S3.** Vasculogenic mimicry-related genes.

**Additional file 6: Table S4.** Differentially expressed VM-related genes (VMDEGs) between normal and tumor samples.

**Additional file 7: Supplementary Fig. 3.** The KEGG and GO analysis of VMDEGs between normal and tumor samples. (A-B) KEGG pathway analysis. (C–D) GO enrichment analysis.

**Additional file 8: Supplementary Fig. 4.** The plots of the Schoenfeld Residuals against the transformed time for model genes.

**Additional file 9: Table S5.** Samples and clinical information in training and testing sets.

**Additional file 10: Supplementary Fig. 5.** Distribution of the risk score, survival status of patients, and mRNA expression heatmap. (A–C) Distribution of the three-gene risk score: (A) Training set; (B) Testing set; (C) Validation set. (D–F) Distribution of the survival status of patients: (D) Training set; (E) Testing set; (F) Validation set. (G–I) Heatmap of the expression of the three-gene signature. (G) Training set; (H) Testing set; (I) Validation set.

**Additional file 11: Table S6.** DEGs between between the high- and low-risk groups.

**Additional file 12: Supplementary Fig. 6.** Functional enrichment analysis of DEGs between the high- and low-risk groups. (A-B) KEGG pathway analysis. (C–D) GO enrichment analysis. (E–F) Gene set enrichment analysis.

**Additional file 13: Supplementary Fig. 7.** Abundance of infiltrated immune cells between the high- and low-risk groups. (A) Differences in immune cell infiltration among LSCC samples. (B) Correlation between immune cells in LSCC samples.

**Additional file 14: Table S7.** Patients with LSCC were divided into two subtypes.

**Additional file 15: Table S8. **Acquisition of the VM score in LSCC using the ssGSEA algorithm.

**Additional file 16: Supplementary Fig. 8.** KM survival analysis of *AGPS* and *EPO* on *OS* in LSCC. KM survival analysis of *AGPS*(A) and *EPO* (B)on *OS* in LSCC.

**Additional file 17: Table S9.** Detailed data of the Venn diagram for screening core genes.

**Additional file 18: Supplementary Fig. 9.** Multivariate analysis of OS for the three genes in LSCC. (A) Multivariate analysis for *TWIS1* on OS. (B) Multivariate analysis for *EPO* on OS. (C) Multivariate analysis for *AGPS* on OS.

**Additional file 19: Supplementary Fig. 10.** Spearman’s correlation analysis was conducted between the expression of *BMP2* and the pathway score. (A) Correlation analysis of the expression of EMT markers with *BMP2*. (B) Correlation analysis of angiogenesis with *BMP2*. (C) Correlation analysis of the PI3K_AKT_mTOR_pathway with *BMP2*.

**Additional file 20: Table S10.** Detailed data of the Venn diagram for screening transcription factors.

**Additional file 21: Supplementary Fig. 11.***RUNX1* is highly expressed in LSCC tissues and correlated with poor clinical features of the tumor. (A-B) *RUNX1* staining and comparison of IHC scores in LSCC tumor and normal tissues. (C-D) *RUNX1* staining and comparison of IHC scores in I-III stage and IV stage patients. *P < 0.05, ****P < 0.0001, Student’s t-test.

**Additional file 22.**

## Data Availability

No datasets were generated or analysed during the current study.

## Abbreviations

BMP: Bone morphogenetic protein
ChIP: Chromatin immunoprecipitation
CNV: Copy number variation
CSC: Cancer stem cells
EMT: Epithelial-mesenchymal transition
FC: Fold change
GO: Gene ontology
GSEA: Gene set enrichment analysis
GSVA: Gene set variation analysis
GTEx: Genotype-tissue expression
HNSCC: Head and neck squamous cell carcinoma
IF: Immunofluorescent
IHC: Immunohistochemistry
KEGG: Kyoto encyclopaedia of genes and genomes
LSCC: Laryngeal squamous cell carcinoma
NSCLC: Non-small cell lung cancer
OS: Overall survival
PAS: Periodic acid-schiff
PBS: Phosphate buffered saline
RNA-seq: RNA sequencing
ROC: Receiver operating curve
TCGA: The cancer genome atlas
TGF-β: Transforming growth factor- β
TMB: Tumor mutational burden
VM: Vasculogenic mimicry
WB: Western blotting
