# Supplementary material for: RUNX1-BMP2 promotes vasculogenic mimicry in laryngeal squamous cell carcinoma via activation of the PI3K-AKT signaling pathway
Source: Cell Commun Signal. 2024 Apr 12;22:227. doi: 10.1186/s12964-024-01605-x (PMC11010429; doi:10.1186/s12964-024-01605-x)

STR 基因型检测报告

样本名称：TU212

检测方法： 用 Axygen 的基因组抽提试剂盒提取 DNA，采用 20- STR 扩增方案扩增，在 ABI 3730XL 型遗传分析仪上对 STR 位点和性别基因 Amelogenin 进行检测。

检测结果： 该株细胞 DNA 分型在细胞系检索中找到**完全匹配**的细胞系，EXPASY 数据库显示细胞名为 Tu 212，细胞号对应 CVCL_4915。本次检测在该细胞系中**没有发现多等位基因**。细胞匹配值：1.0

STR 数据库比对分析： 待测细胞的 STR 位点和 Amelogenin 位点的基因分型结果与收录于 ExPASY,ATCC, DSMZ, JCRB 和 RIKEN 数据库的 2455 个细胞系 STR 数据进行比对，如果待检测细胞未收录于以上细胞库或这是自行建立的新细胞系将无法进行比对，用户需根据细胞分型结果自行与其他数据库进行比对。

分型结果：

Loci	送检细胞 STR 信息			细胞库细胞 STR 信息		
	送检细胞名：TU212			细胞库细胞名： Tu 212		
	Allele1	Allele2	Allele3	Allele1	Allele2	Allele3
D5S818	12	13		12	13	
D13S317	12	12		12	12	
D7S820	10	10		10	10	
D16S539	8	9		8	9	
VWA	15	16		15	16	
TH01	8	8		8	8	
AMEL	X	X		X	X	
TPOX	10	11		10	11	
CSF1PO	12	13		12	13	
D12S391	17	19				
FGA	18.2	18.2				
D2S1338	21	21				
D21S11	28	29				
D18S51	14	14				
D8S1179	13	13				
D3S1358	14	14				
D6S1043	15	17				
PENTAE	12	12				
D19S433	13	13				
PENTAD	14	14				

分型图谱：

Applied Biosystems

GeneMapper 4.0

Cell Line Authentication 1129 1201

Sample File	Sample Name	Panel	OS	SQ
53_E07.fsa	Tu212	STR Profile 1-human		

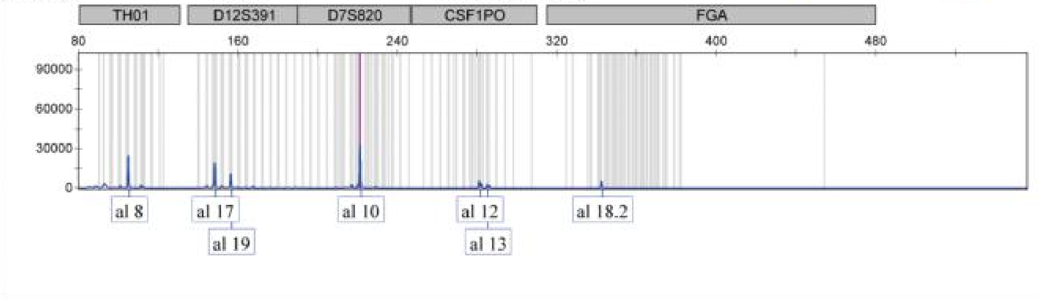

Sample File	Sample Name	Panel	OS	SQ
53_E07.fsa	Tu212	STR Profile 1-human		

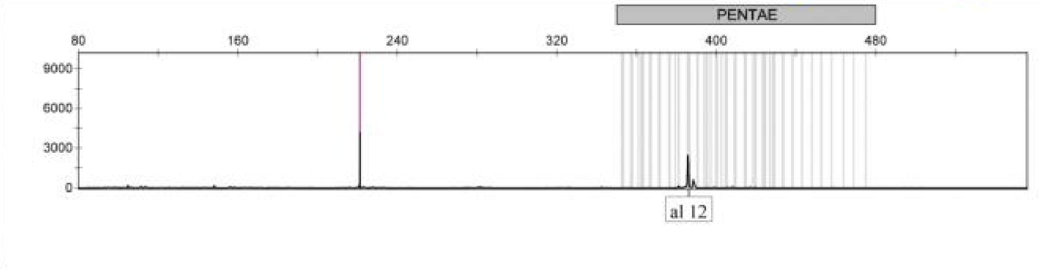

Sample File	Sample Name	Panel	OS	SQ
93_E12.fsa	Tu212	STR Profile 2-human		

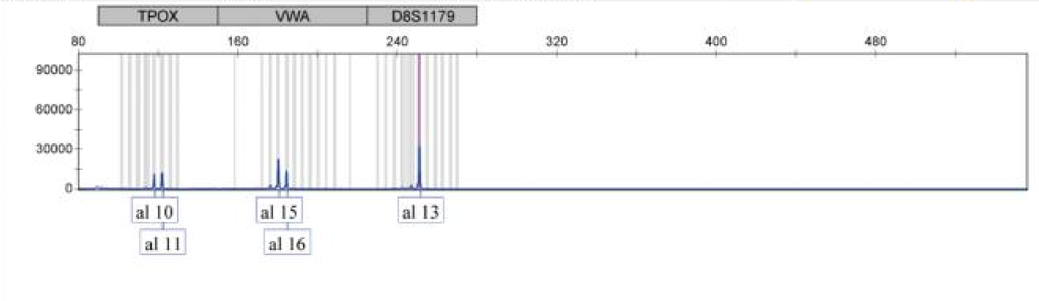

Cell Line Authentication 1129 1201

Sample File	Sample Name	Panel	OS	SQ
93_E12.fsa	Tu212	STR Profile 2-human		

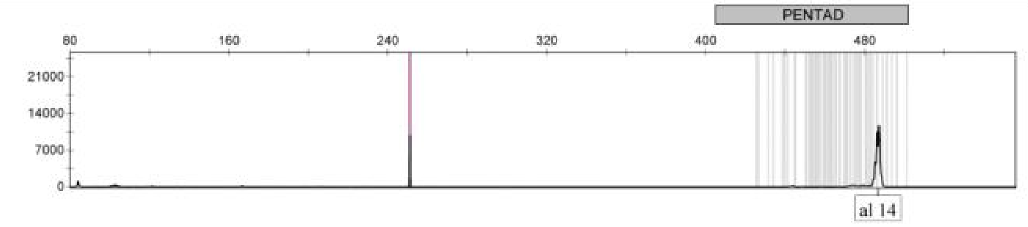

21_E03.fsa	Tu212	STR Profile 3-human		

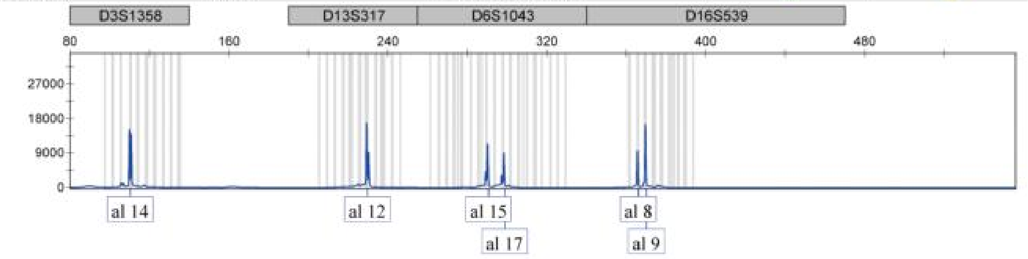

21_E03.fsa	Tu212	STR Profile 3-human		

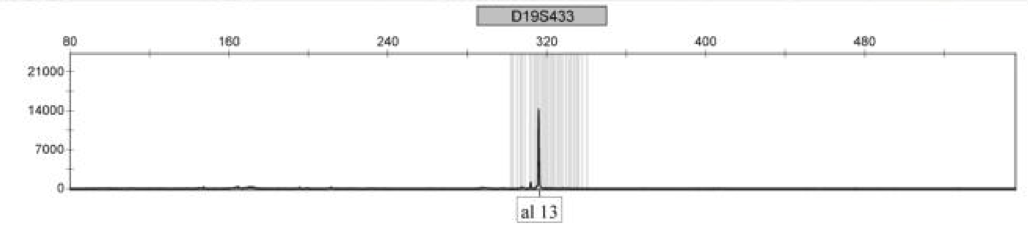

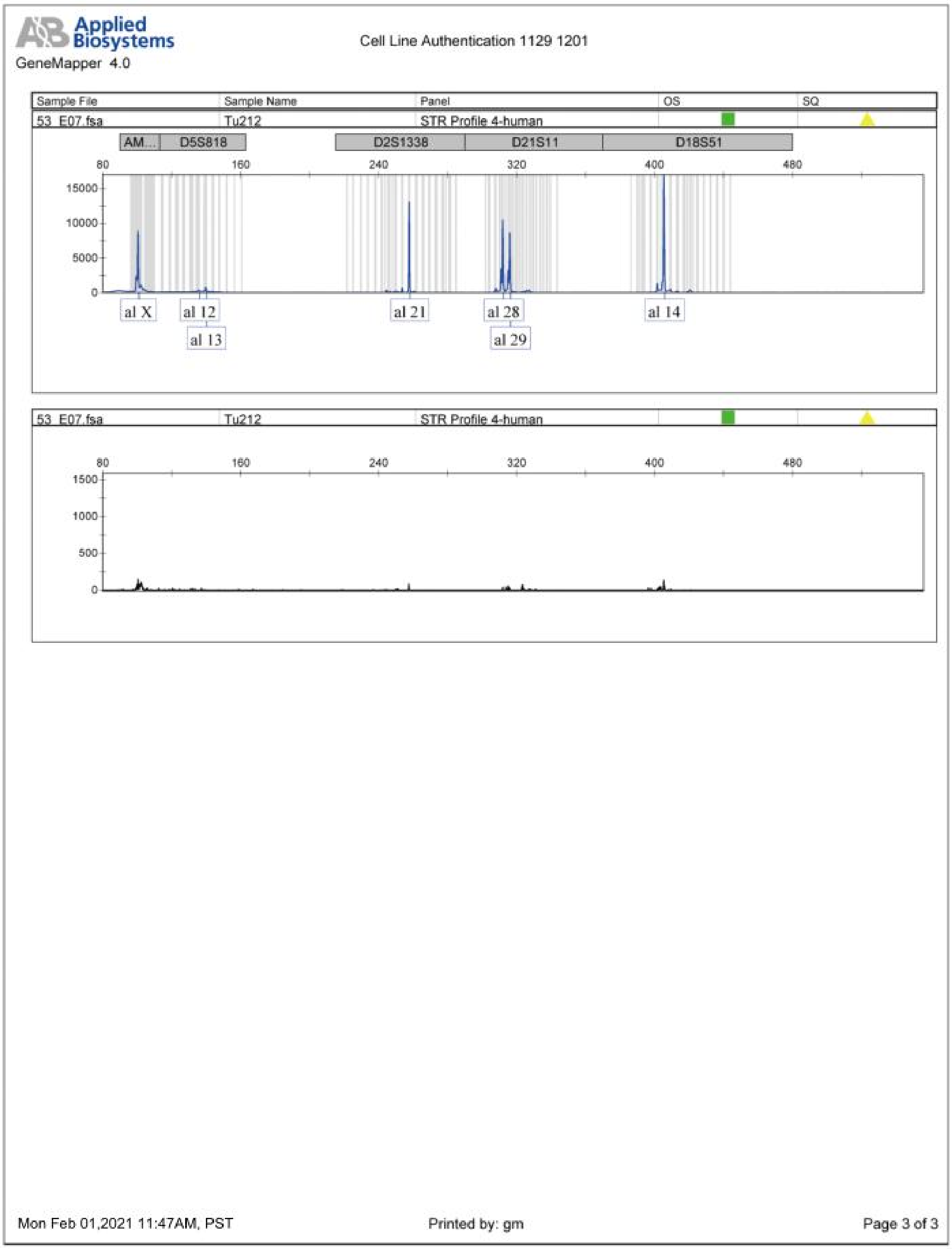

备注:

1.根据国际细胞鉴定委员会(ICLAC)制定的细胞 STR 鉴定标准,匹配度≥80%即可认为该细胞系正确,匹配度 < 80%则说明该细胞系的来源需要被怀疑。

2. 图谱有效峰位真实的 PCR 条带,小峰和非特异性条带在计算中忽略不计。

3. 国家实验细胞资源共享平台,数据库入口 http://www.cellresource.cn /。

细胞遗传质量鉴定检测

Cell Line Authentication Service

STR 基因型检测报告

送检单位：赛百慷

检品名称：细胞系

委托单位：上海翼和应用生物技术有限公司

报告日期：2019-10-08

上海翼和应用生物技术有限公司 联系地址：上海市漕河泾开发区桂平路 471 号桂果园 8 号楼 4F 室
网址：http://www.biowing.com.cn/邮箱：biowing@vip.163.com 服务热线：021-33559491

报 告 说 明

1. 本报告只对送检的来样负责。
2. 检验报告上的检验结果和检验单位名称，未经同意不得用于广告、评优及商业宣传。
3. 对本报告有异议，请于收到报告之日起十五日内以书面方式提出，逾期不予受理。
4. 对纸质检验报告涂改、增删，或未加盖检验单位印章的复印件均无效。

样 品 信 息

样品编号：

客户样本编号	公司编号
TU686	20190923-02

样品数量： 1

样品性状： 细胞系

检测项目： STR

送检单位： 赛百慷

检测方法： 用 Axygen 的基因组抽提试剂盒提取 DNA，采用 20- STR 扩增方案扩增，在 ABI 3730XL 型遗传分析仪上对 STR 位点和性别基因 Amelogenin 进行检测。

上海翼和应用生物技术有限公司 联系地址：上海市漕河泾开发区桂平路 471 号桂果园 8 号楼 4F 室
网址：http://www.biowing.com.cn/邮箱：biowing@vip.163.com 服务热线：021-33559491

检 测 结 果

(一)检验基本情况

	多等位基因	匹配细胞系	细胞库	EV 值	匹配说明
20190923-02	无	-	DSMZ	-	无匹配

样本基因型检验结果

- 多等位基因指三等位及以上基因现象。
- 本次检测各细胞分型结果良好。

(二)各样本描述

- 20190923-02：该株细胞 DNA 分型在细胞系检索中**没有找到匹配**的细胞系，本次检测在该细胞系中**没有发现多等位基因**。（该细胞系未发现多等位基因、未发现交叉污染，细胞系本身无异常，因数据库未收录 TU686 相关 STR 信息无法匹配，若为发表论文用，提交该数据给杂志即可）

  备注：待测细胞系与收录于 ATCC, DSMZ, JCRB 和 RIKEN 数据库的细胞系 STR 数据进行比对，未收录于以上细胞库的细胞系将无法匹配。

（三）样本分型结果

细胞 20190923-02 的 STR 位点和 Amelogenin 位点的基因分型结果						
Loci	送检细胞 STR 信息			细胞库细胞 STR 信息		
	送检细胞名：TU686			细胞库细胞名：		
	Allele1	Allele2	Allele3	Allele1	Allele2	Allele3
D5S818	12	12				
D13S317	12	12				
D7S820	10	10				
D16S539	8	9				
VWA	15	16				
TH01	8	8				
AMEL	X	X				
TPOX	10	11				
CSF1PO	12	13				
D12S391	17	19				
FGA	18.2	18.2				
D2S1338	21	21				
D21S11	28	29				
D18S51	14	14				
D8S1179	13	13				
D3S1358	14	14				
D6S1043	15	17				
PENTAE	12	12				
D19S433	13	13				
PENTAD	14	14				

其 他 说 明

(一)分型方案及位点分布

	方案 1	方案 2	方案 3	方案 4
1	TH01	TPOX	D3S1358	AMEL
2	D12S391	VWA	D13S317	D5S818
3	D7S820	D8S1179	D6S1043	D2S1338
4	CSF1PO	PENTAD	D16S539	D21S11
5	FGA		D19S433	D18S51
6	PENTAE			

实验方案及位点

(二)STR 数据库比对

本公司采用 DSMZ tools 进行细胞系比对，其中包含来自于 ATCC, DSMZ, JCRB 和 RIKEN 数据库的 2455 个细胞系 STR 数据。如果待检测细胞未收录于以上细胞库或这是自行建立的新细胞系将无法进行比对，用户需根据细胞分型结果自行与其他数据库进行比对。

(三)文献引用参考

1．Authentication testing of HEK 293T and HeLa cell lines have been performed by Shanghai Biowing Applied Biotechnology Co.,Ltd via STR profiling. STR profiles match the standards recommended for HEK 293T and HeLa cell lines authentication

2．AGS, NCI-N87, HGC-27 and HEK293 were STR-authenticated on Dec. 8, 2015 by Shanghai Biowing Applied Biotechnology Co. LTD, Shanghai, China

主要实验人员：张佳男

复核人: 龚礼瑾

负责人：陈轶群

上海翼和应用生物技术有限公司 联系地址：上海市漕河泾开发区桂平路 471 号桂果园 8 号楼 4F 室
网址：http://www.biowing.com.cn/邮箱：biowing@vip.163.com 服务热线：021-33559491

签发日期：2019 年 10 月 08 日

细胞遗传质量鉴定检测

Cell Line Authentication Service

STR 基因型检测报告

送检单位：赛百慷

检品名称：细胞系

委托单位：上海翼和应用生物技术有限公司

报告日期：2022-03-04

报 告 说 明

1. 本报告只对送检的来样负责。
2. 检验报告上的检验结果和检验单位名称，未经同意不得用于广告、评优及商业宣传。
3. 对本报告有异议，请于收到报告之日起十五日内以书面方式提出，逾期不予受理。
4. 对纸质检验报告涂改、增删，或未加盖检验单位印章的复印件均无效。

样 品 信 息

样品编号：

客户样本编号	公司编号
hacat	20220225-06

样品数量： 1

样品性状： 细胞系

检测项目： STR

送检单位： **赛百慷**

检测方法： 用 Axygen 的基因组抽提试剂盒提取 DNA，采用 21- STR 扩增方案扩增，在 ABI 3730XL 型遗传分析仪上对 STR 位点和性别基因 Amelogenin 进行检测。

检 测 结 果

(一)检验基本情况

公司编号	多等位基因	匹配细胞系	细胞库	EV 值	匹配说明
20220225-06	无	HACAT	DSMZ	1.0	完全匹配

样本基因型检验结果

- 多等位基因指三等位及以上基因现象。
- 本次检测各细胞分型结果良好。

(二)各样本描述

- 20220225-06：该株细胞 DNA 分型在细胞系检索中找到**完全匹配**的细胞系，DSMZ 数据库显示细胞名为 HACAT，细胞号对应 771。本次检测在该细胞系中**没有发现多等位基因**。

  备注：待测细胞系与收录于 ATCC, DSMZ, JCRB 和 RIKEN 数据库的细胞系 STR 数据进行比对，未收录于以上细胞库的细胞系将无法匹配。

（三）样本分型结果

细胞 20220225-06 的 STR 位点和 Amelogenin 位点的基因分型结果						
Loci	送检细胞 STR 信息			细胞库细胞 STR 信息		
	送检细胞名：hacat			细胞库细胞名：HACAT		
	Allele1	Allele2	Allele3	Allele1	Allele2	Allele3
D5S818	12	12		12	12	
D13S317	10	12		10	12	
D7S820	9	11		9	11	
D16S539	9	12		9	12	
VWA	16	17		16	17	
TH01	9.3	9.3		9.3	9.3	
AMEL	X	X		X	X	
TPOX	11	12		11	12	
CSF1PO	9	11		9	11	
D12S391	18	23				
FGA	23.2	23.2				
D2S1338	24.2	24.2				
D21S11	30.2	30.2				
D18S51	12	12				
D8S1179	14	14				
D3S1358	16	16				
D6S1043	12	19				
PENTAE	6.3	12				
D19S433	13	14				
PENTAD	13	13				
D1S1656	11	12				

其 他 说 明

(一)分型方案及位点分布

	方案 1	方案 2	方案 3	方案 4
1	D3S1358	D8S1179	D19S433	AMEL
2	VWA	D21S11	TH01	D1S1656
3	D7S820	D16S539	D13S317	D5S818
4	CSF1PO	D2S1338	TPOX	D12S391
5	PENTAE	PENTAD	D18S51	FGA
6			D6S1043	

实验方案及位点

(二)STR 数据库比对

本公司采用 DSMZ tools 进行细胞系比对，其中包含来自于 ATCC, DSMZ, JCRB 和 RIKEN 数据库的 2455 个细胞系 STR 数据。如果待检测细胞未收录于以上细胞库或这是自行建立的新细胞系将无法进行比对，用户需根据细胞分型结果自行与其他数据库进行比对。

(三)文献引用参考

1．Authentication testing of HEK 293T and HeLa cell lines have been performed by Shanghai Biowing Applied Biotechnology Co.,Ltd via STR profiling. STR profiles match the standards recommended for HEK 293T and HeLa cell lines authentication
2．AGS, NCI-N87, HGC-27 and HEK293 were STR-authenticated on Dec. 8, 2015 by Shanghai Biowing Applied Biotechnology Co. LTD, Shanghai, China

主要实验人员：张佳男
复核人: 张晨茜
负责人： 白杨

签发日期：2022-03-04

Applied Biosystems
GeneMapper ID v3.2

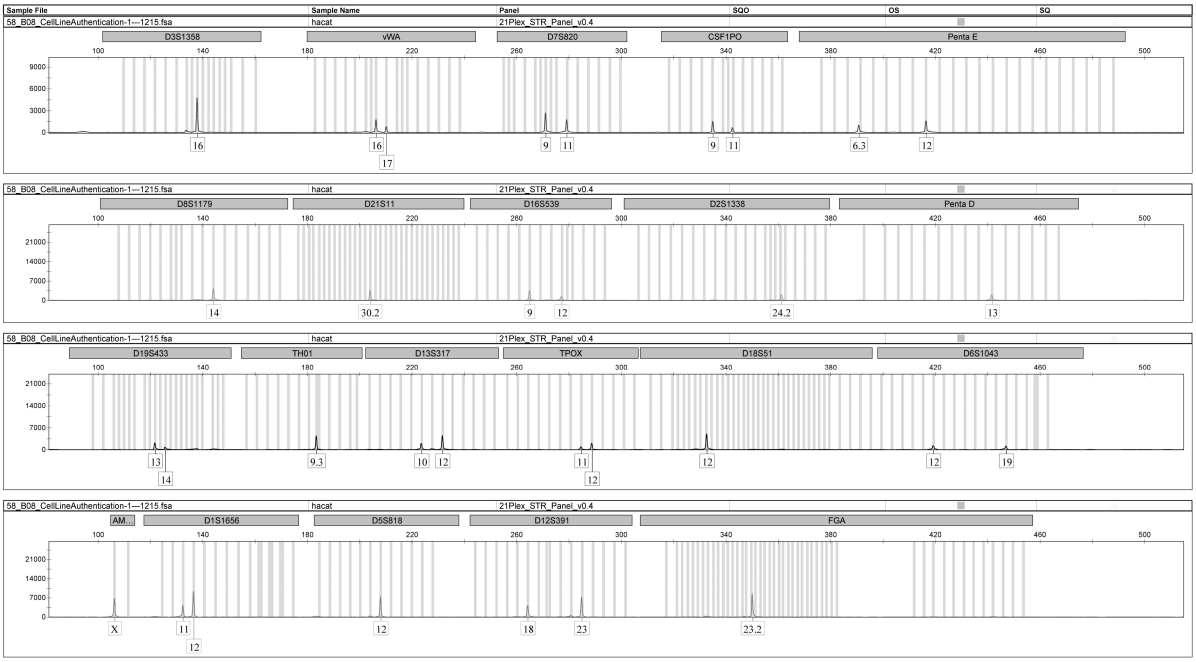

Supplement: Supplementary file 1 — Additional file 1: Supplementary Fig. 1. STR identification certificates of the three cell lines involved in this study, hacat, tu212, and tu686. [file 12964_2024_1605_MOESM1_ESM.pdf]
