# Supplementary figures and images for: RUNX1-BMP2 promotes vasculogenic mimicry in laryngeal squamous cell carcinoma via activation of the PI3K-AKT signaling pathway
Source: Cell Commun Signal. 2024 Apr 12;22:227. doi: 10.1186/s12964-024-01605-x (PMC11010429; doi:10.1186/s12964-024-01605-x)

Mycoplasma testing

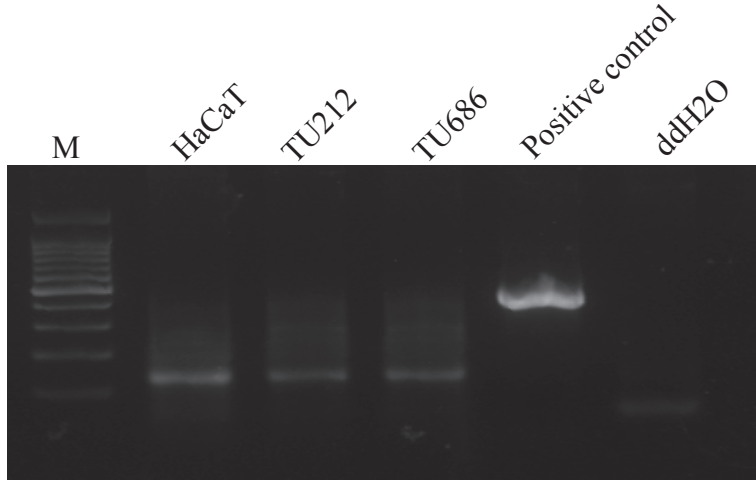

Supplement: Supplementary file 2 — Additional file 2: Supplementary Fig. 2. Three cells were free of mycoplasma contamination, as determined by MycoProbe mycoplasma detection kit. [file 12964_2024_1605_MOESM2_ESM.pdf]

A

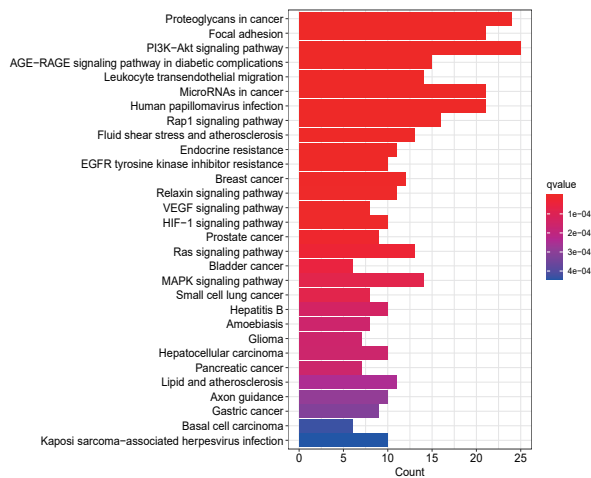

B

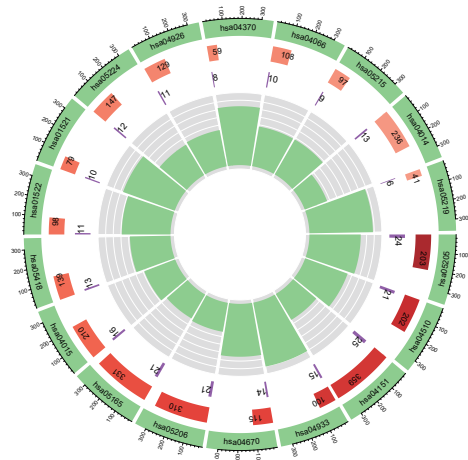

C

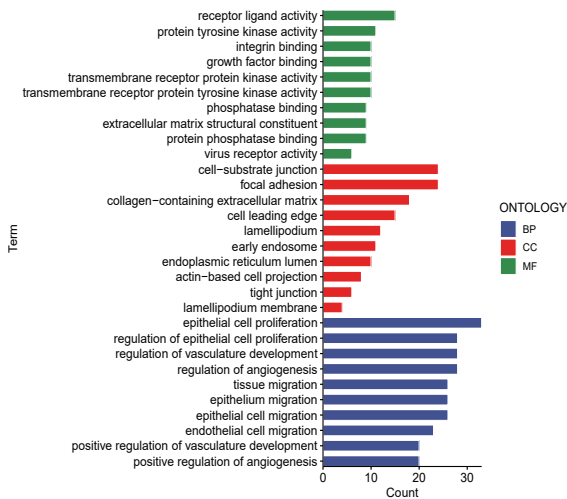

D

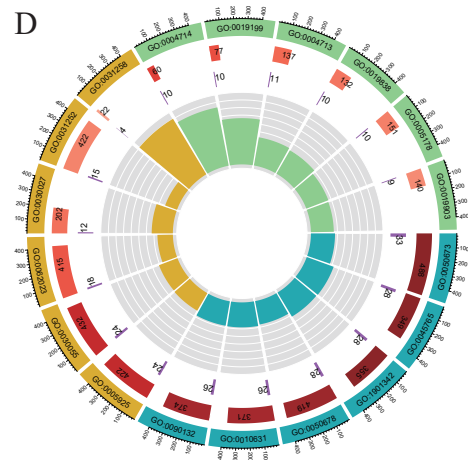

Supplement: Supplementary file 7 — Additional file 7: Supplementary Fig. 3. The KEGG and GO analysis of VMDEGs between normal and tumor samples. (A-B) KEGG pathway analysis. (C–D) GO enrichment analysis. [file 12964_2024_1605_MOESM7_ESM.pdf]

Global Schoenfeld Test p: 0.7707

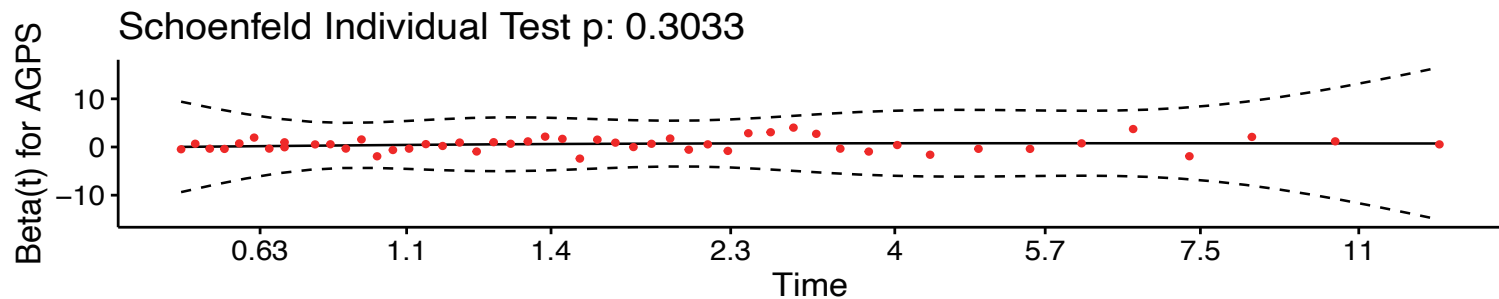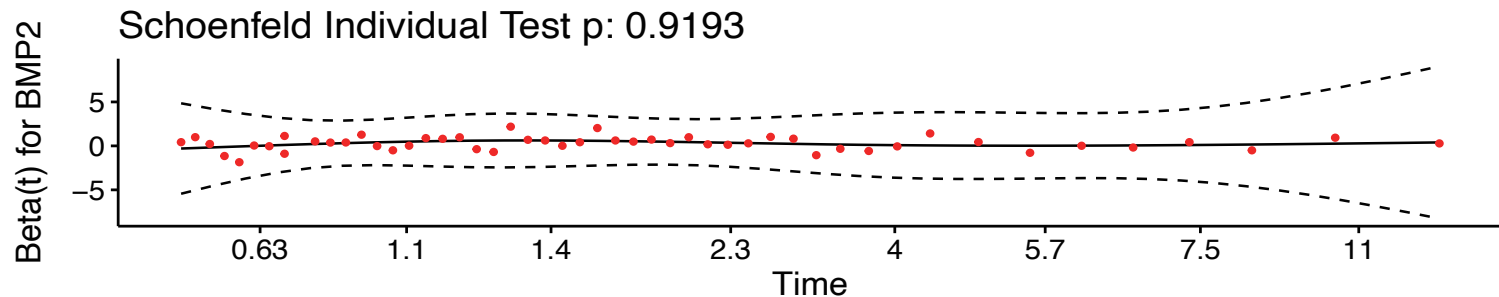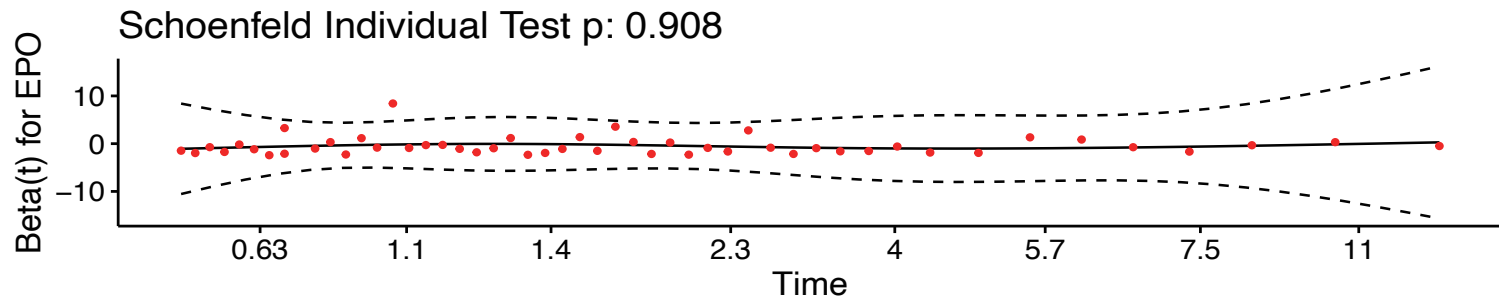

Supplement: Supplementary file 8 — Additional file 8: Supplementary Fig. 4. The plots of the Schoenfeld Residuals against the transformed time for model genes. [file 12964_2024_1605_MOESM8_ESM.pdf]

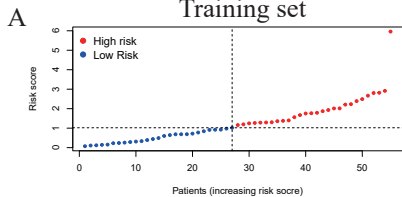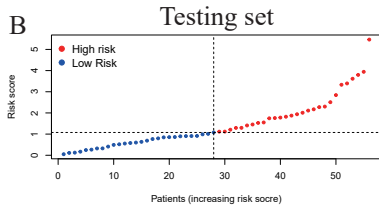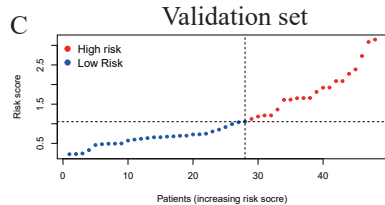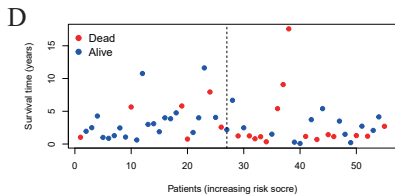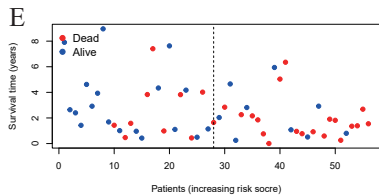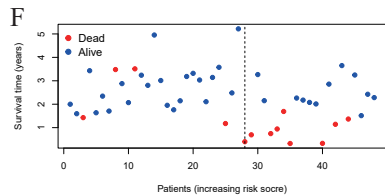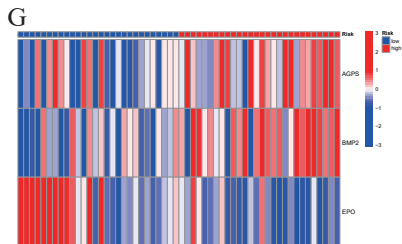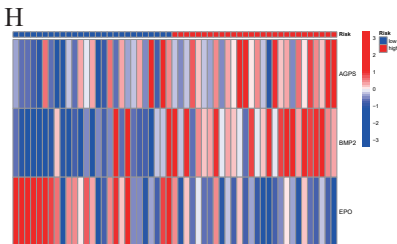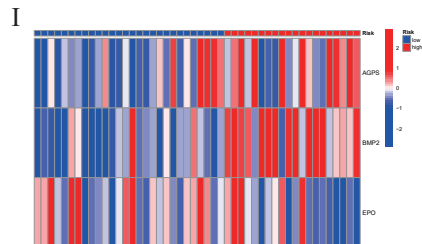

Supplement: Supplementary file 10 — Additional file 10: Supplementary Fig. 5. Distribution of the risk score, survival status of patients, and mRNA expression heatmap. (A–C) Distribution of the three-gene risk score: (A) Training set; (B) Testing set; (C) Validation set. (D–F) Distribution of the survival status of patients: (D) Training set; (E) Testing set; (F) Validation set. (G–I) Heatmap of the expression of the three-gene signature. (G) Training set; (H) Testing set; (I) Validation set. [file 12964_2024_1605_MOESM10_ESM.pdf]

A

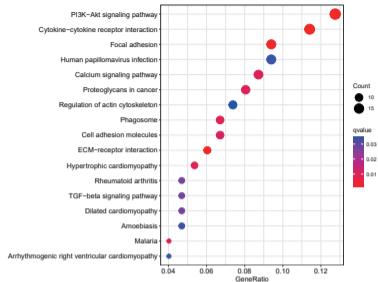

C

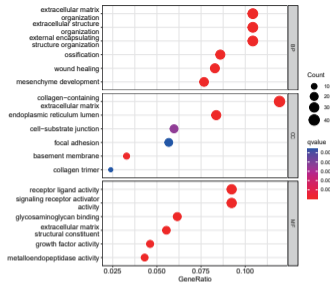

E

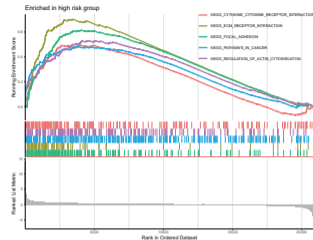

B

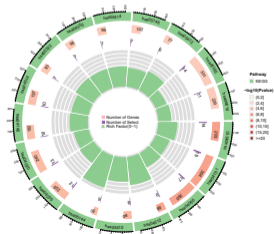

D

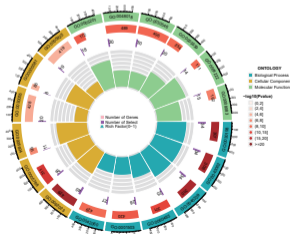

F

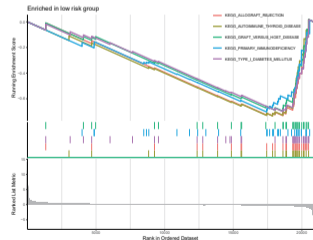

Supplement: Supplementary file 12 — Additional file 12: Supplementary Fig. 6. Functional enrichment analysis of DEGs between the high- and low-risk groups. (A-B) KEGG pathway analysis. (C–D) GO enrichment analysis. (E–F) Gene set enrichment analysis. [file 12964_2024_1605_MOESM12_ESM.pdf]

A

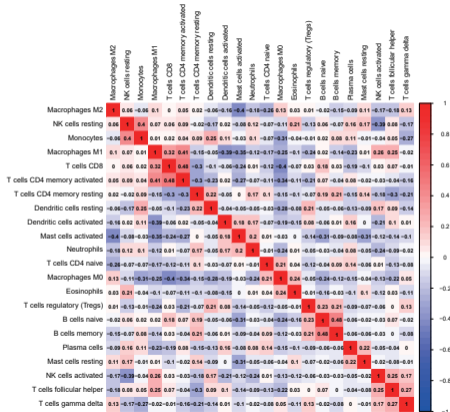

B

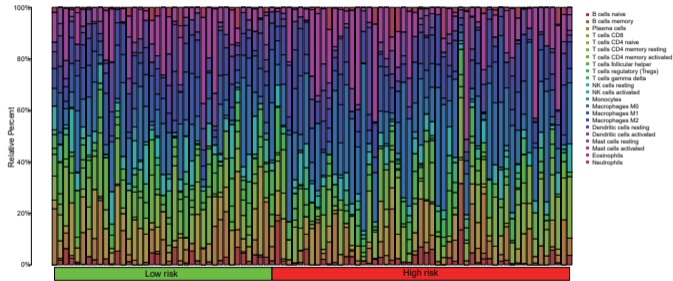

Supplement: Supplementary file 13 — Additional file 13: Supplementary Fig. 7. Abundance of infiltrated immune cells between the high- and low-risk groups. (A) Differences in immune cell infiltration among LSCC samples. (B) Correlation between immune cells in LSCC samples. [file 12964_2024_1605_MOESM13_ESM.pdf]

A

AGPS

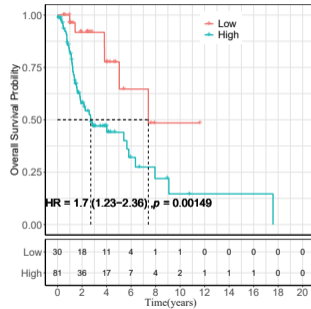

B

EPO

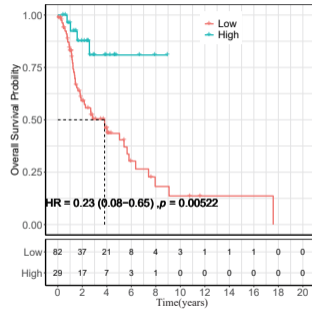

Supplement: Supplementary file 16 — Additional file 16: Supplementary Fig. 8. KM survival analysis of AGPS and EPO on OS in LSCC. KM survival analysis of AGPS(A) and EPO (B)on OS in LSCC. [file 12964_2024_1605_MOESM16_ESM.pdf]

A

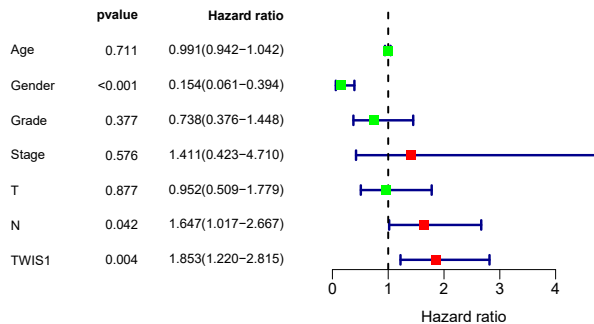

B

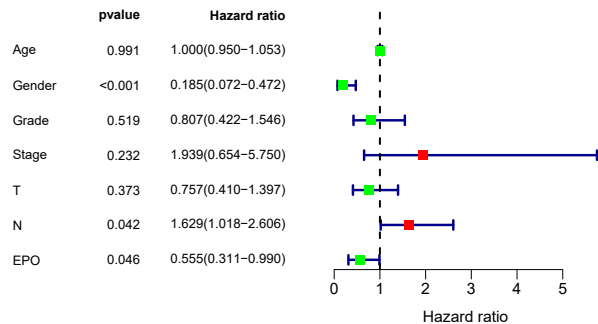

C

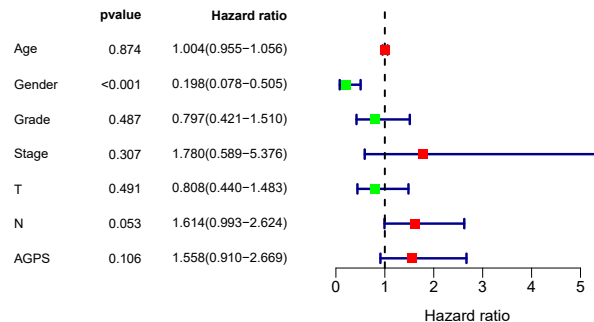

Supplement: Supplementary file 18 — Additional file 18: Supplementary Fig. 9. Multivariate analysis of OS for the three genes in LSCC. (A) Multivariate analysis for TWIS1 on OS. (B) Multivariate analysis for EPO on OS. (C) Multivariate analysis for AGPS on OS. [file 12964_2024_1605_MOESM18_ESM.pdf]

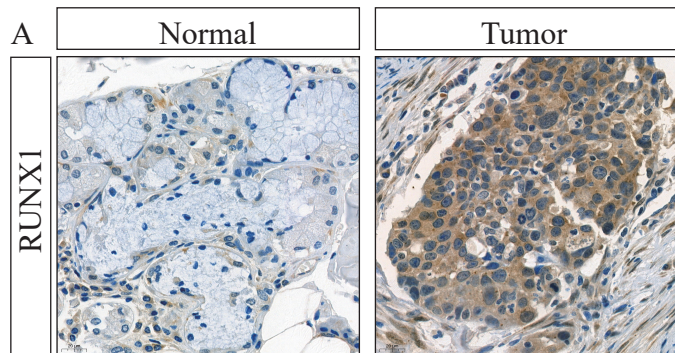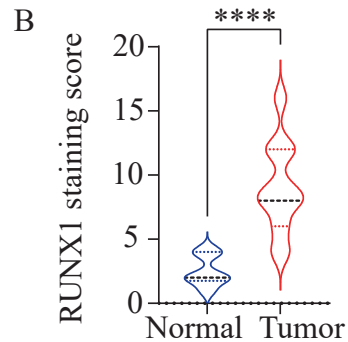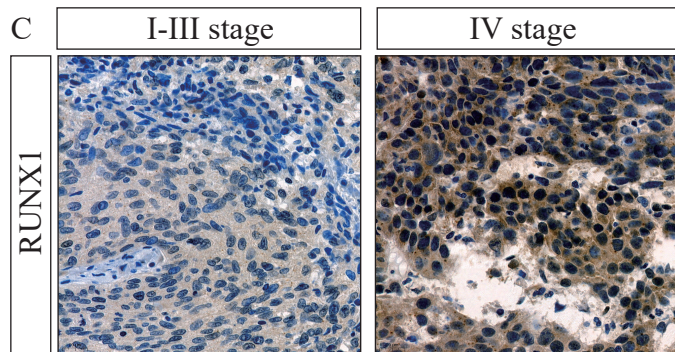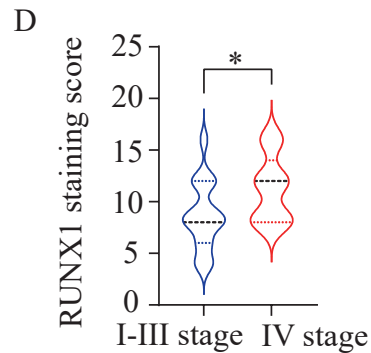

Supplement: Supplementary file 21 — Additional file 21: Supplementary Fig. 11.RUNX1 is highly expressed in LSCC tissues and correlated with poor clinical features of the tumor. (A-B) RUNX1 staining and comparison of IHC scores in LSCC tumor and normal tissues. (C-D) RUNX1 staining and comparison of IHC scores in I-III stage and IV stage patients. *P < 0.05, ****P < 0.0001, Student’s t-test. [file 12964_2024_1605_MOESM21_ESM.pdf]

Figure 6

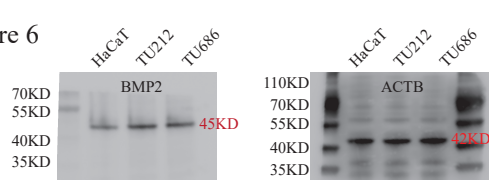

Figure 7

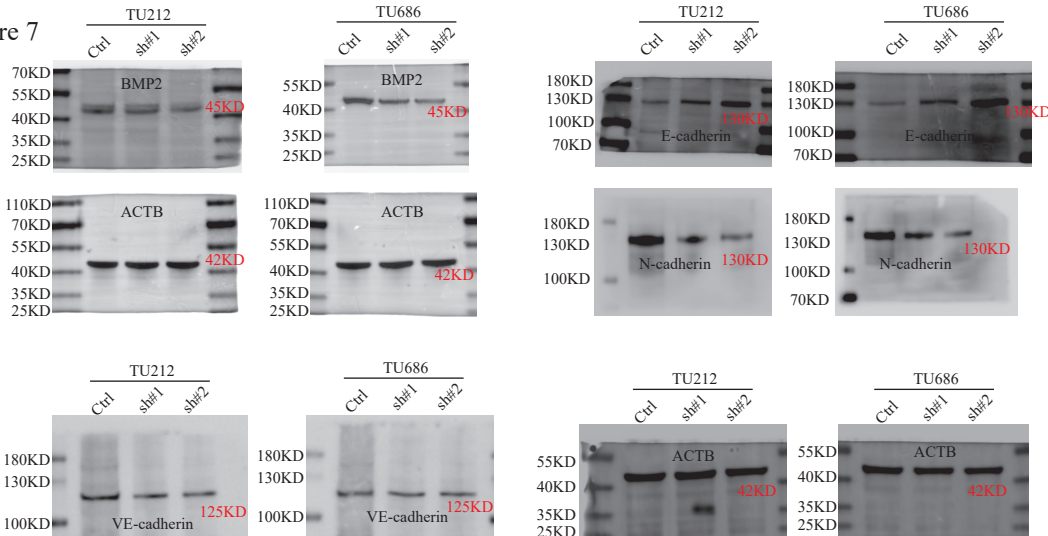

Figure 8

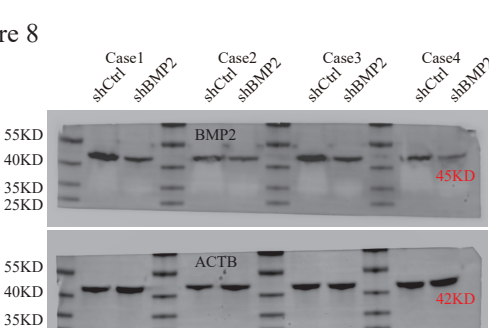

Figure 9

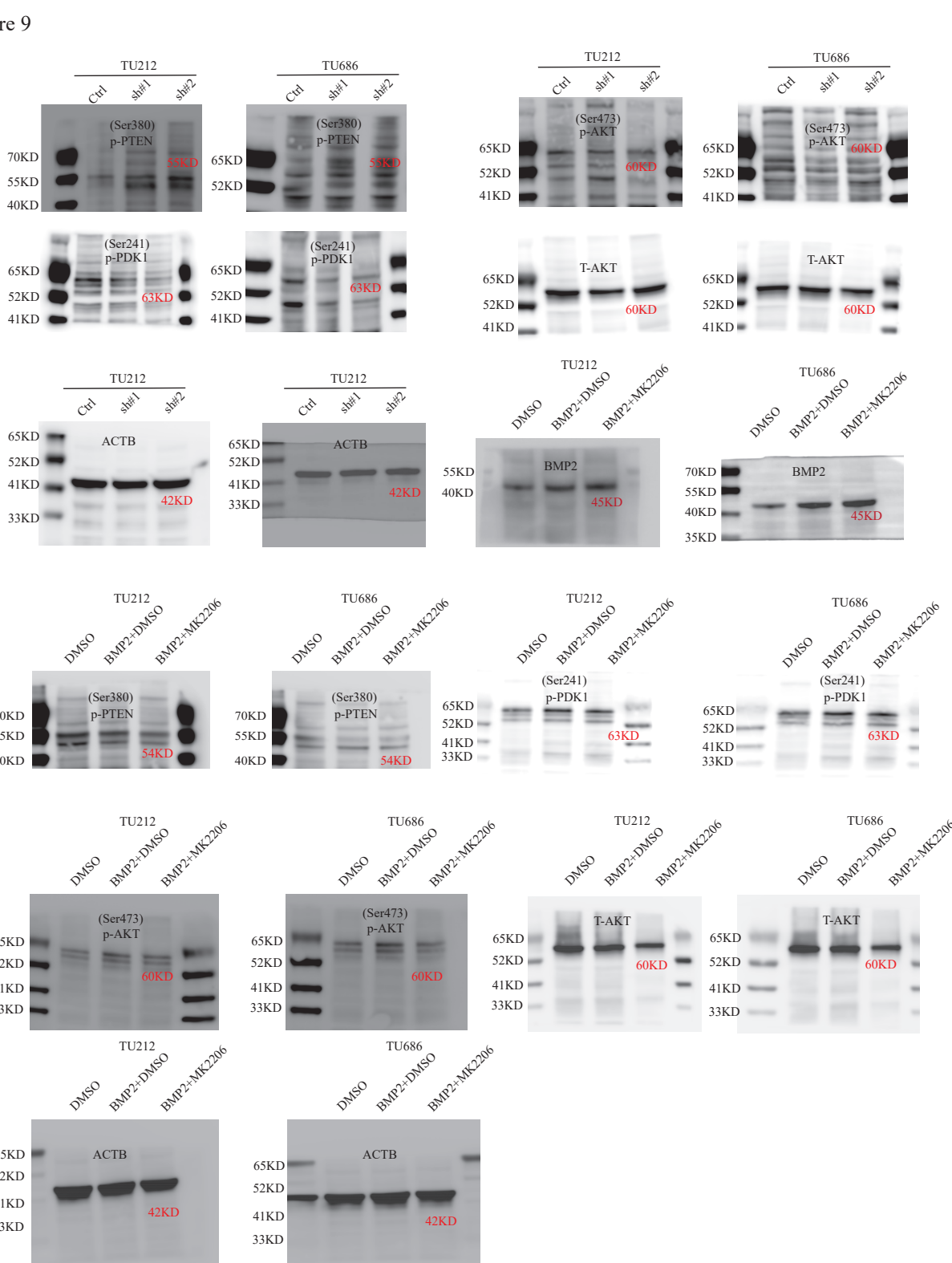

Figure 10

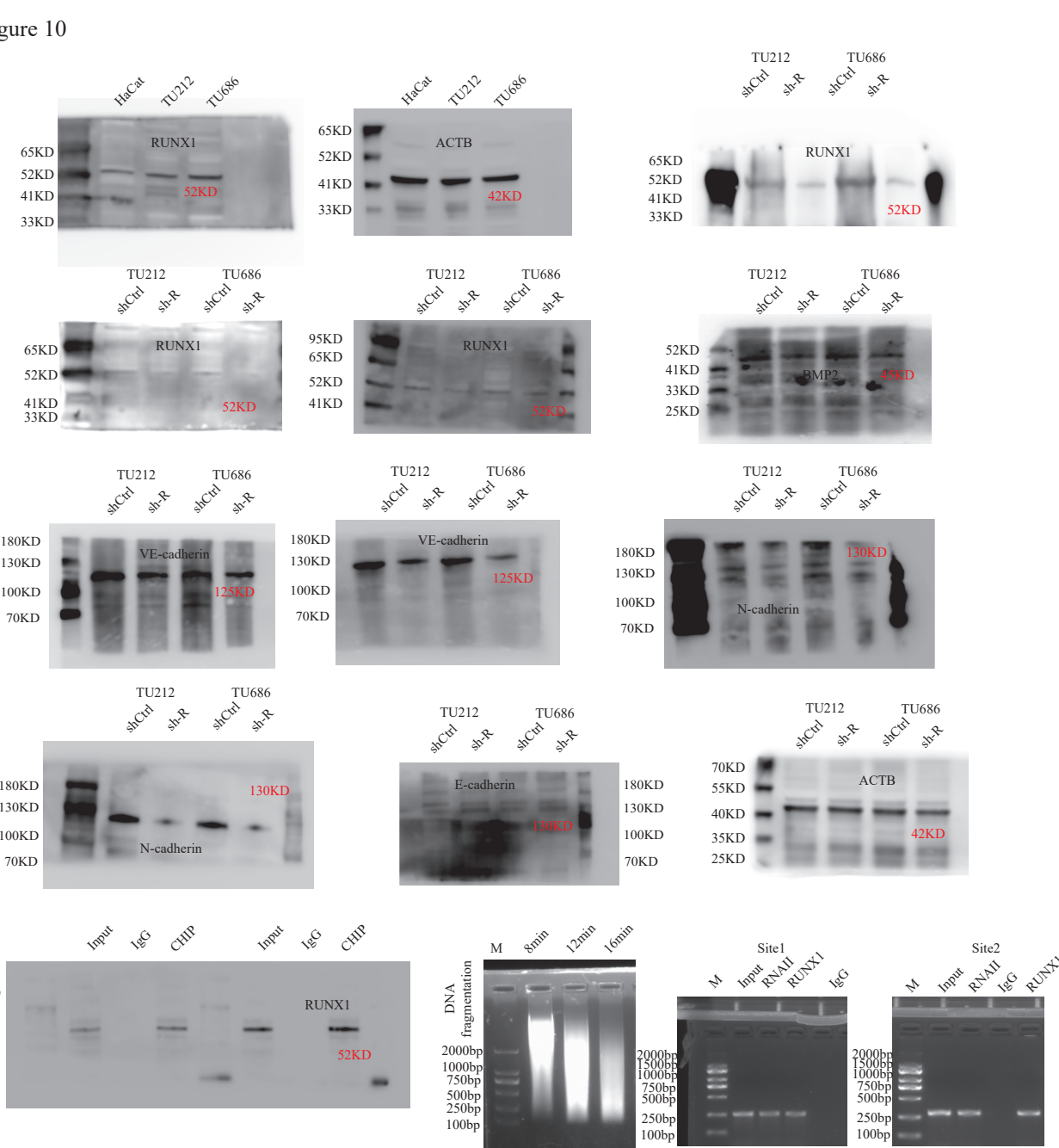

Figure 11

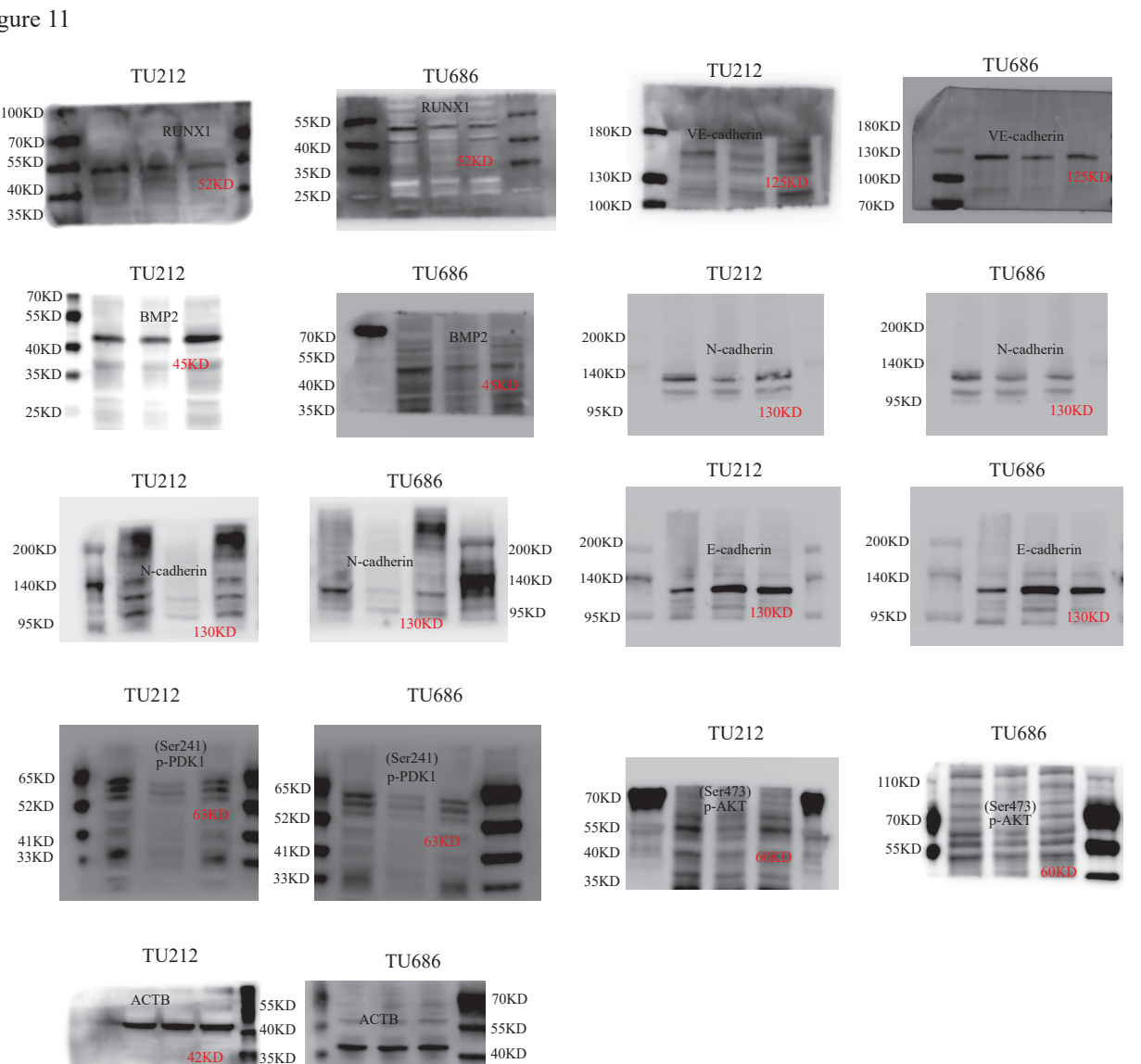

Supplement: Supplementary file 22 — Additional file 22. [file 12964_2024_1605_MOESM22_ESM.pdf]
