# Supplementary material for: RUNX1-BMP2 promotes vasculogenic mimicry in laryngeal squamous cell carcinoma via activation of the PI3K-AKT signaling pathway
Source: Cell Commun Signal. 2024 Apr 12;22:227. doi: 10.1186/s12964-024-01605-x (PMC11010429; doi:10.1186/s12964-024-01605-x)
Supplement: Supplementary file 4 — Additional file 4: Table S2. Wild-type and mutant sequences of binding sites in the BMP2 promoter region. [file 12964_2024_1605_MOESM4_ESM.docx]

BMP2 site1 WT

ATATGTAAATACATACATCTCTATGTATTAATGTTTAAAAACACTCAATT

TCCAGCCTGCTGTTTTCTTTTAATTTTCCTCCTATTCCGGGGAAACAGAA

GCGTGGATCCCACGTCTATGCTATGCCAAAATACGCTGTAATTGAGGTGT

TTTGTTTTGTTTTGTTTTTTGAAATCGTATATTACCGAAAAACTTCAAAC

TGAAAGTTGAATAACGGGCCCAGCGGGGAAATAAGAGGCCAGACCCTGAC

CCTGCATTTGTCCTGGATTTCGCCTCCAGAGTCCCCGCGAGGGTCCGGCG

CGCCAGCTGATCTCTCCTTTGAGAGCAGGGAGTGGAGGCGC

BMP2 site1 MT

ATATGTAAATACATACATCTCTATGTATTAATGTTTAAAAACACTCAATT

TCCAGCCTGCTGTTTTCTTTTAATTTTCCTCCTATTCCGGGGAAACAGAA

GCGTGGATCCCACGTCTATGCTATGCCAAAATACGCTGTAATTGAGGTGT

TTTGTTTTGTTTTGTTTTTTGAAATCGTATAAATGGCTTTTTCTTCAAAC

TGAAAGTTGAATAACGGGCCCAGCGGGGAAATAAGAGGCCAGACCCTGAC

CCTGCATTTGTCCTGGATTTCGCCTCCAGAGTCCCCGCGAGGGTCCGGCG

CGCCAGCTGATCTCTCCTTTGAGAGCAGGGAGTGGAGGCGC

BMP2 site2 WT

CCATGTCACATACATAGACATATTAACCGAAATGTGGCCCTTCGGTTGCA

TATATTCTCATACATGAATATATTTATAGAAATATATGCACATATTTTTG

TATATTGGATATATTTATGTAACTATAAATTTACATGCGTATGGATATGA

AAATAAATGCATACACATTTATGTAAAAAAATTTGTACACATGCATTTAC

ATATGTAAATACATACATCTCTATGTATTAATGTTTAAAAACACTCAATT

TCCAGCCTGCTGTTTTCTTTTAATTTTCCTCCTATTCCGGGGAAACAGAA

BMP2 site2 MT

CCATGTCACATACATAGACATATATTGGCTTTACTGGCCCTTCGGTTGCA

TATATTCTCATACATGAATATATTTATAGAAATATATGCACATATTTTTG

TATATTGGATATATTTATGTAACTATAAATTTACATGCGTATGGATATGA

AAATAAATGCATACACATTTATGTAAAAAAATTTGTACACATGCATTTAC

ATATGTAAATACATACATCTCTATGTATTAATGTTTAAAAACACTCAATT

TCCAGCCTGCTGTTTTCTTTTAATTTTCCTCCTATTCCGGGGAAACAGAA
