# Supplementary material for: RUNX1-BMP2 promotes vasculogenic mimicry in laryngeal squamous cell carcinoma via activation of the PI3K-AKT signaling pathway
Source: Cell Commun Signal. 2024 Apr 12;22:227. doi: 10.1186/s12964-024-01605-x (PMC11010429; doi:10.1186/s12964-024-01605-x)
Supplement: Supplementary file 19 — Additional file 19: Supplementary Fig. 10. Spearman’s correlation analysis was conducted between the expression of BMP2 and the pathway score. (A) Correlation analysis of the expression of EMT markers with BMP2. (B) Correlation analysis of angiogenesis with BMP2. (C) Correlation analysis of the PI3K_AKT_mTOR_pathway with BMP2. [file 12964_2024_1605_MOESM19_ESM.pdf]

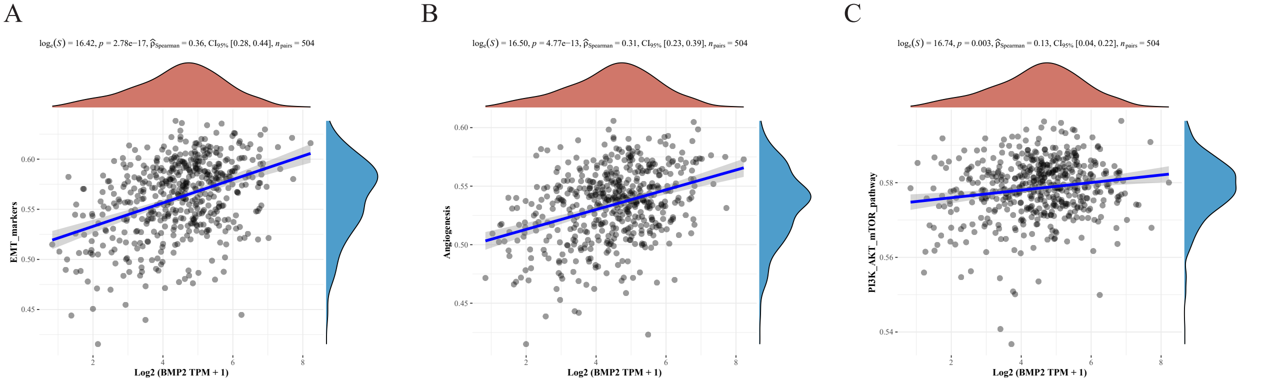
A
log_e(S) = 16.42, p = 2.78e−17, ρ̂_Spearman = 0.36, CI_95% [0.28, 0.44], n_pairs = 504
EMT_markers
Log2 (BMP2 TPM + 1)
B
log_e(S) = 16.50, p = 4.77e−13, ρ̂_Spearman = 0.31, CI_95% [0.23, 0.39], n_pairs = 504
Angiogenesis
Log2 (BMP2 TPM + 1)
C
log_e(S) = 16.74, p = 0.003, ρ̂_Spearman = 0.13, CI_95% [0.04, 0.22], n_pairs = 504
PI3K_AKT_mTOR_pathway
Log2 (BMP2 TPM + 1)
